# A value of information framework for assessing the trade-offs associated with uncertainty, duration, and cost of chemical toxicity testing

**DOI:** 10.1111/risa.13931

**Published:** 2022-04-22

**Authors:** Shintaro Hagiwara, Greg M. Paoli, Paul S. Price, Maureen R. Gwinn, Annette Guiseppi-Elie, Patrick J. Farrell, Bryan J. Hubbell, Daniel Krewski, Russell S. Thomas

**Affiliations:** 1 Risk Sciences International, Ottawa, Canada; 2 School of Mathematics and Statistics, Carleton University, Ottawa, Canada; 3 Center for Compuational Toxicology and Exposure, Office of Research and Development, US Environmental Protection Agency, Research Triangle Park, North Carolina, USA; 4 Office of Research and Development, US Environmental Protection Agency, Research Triangle Park, North Carolina, USA; 5 Air, Climate, and Energy Research Program, Office of Research and Development, US Environmental Protection Agency, Research Triangle Park, North Carolina, USA; 6 McLaughlin Centre for Population Health Risk Assessment, University of Ottawa, Ottawa, Canada

**Keywords:** cost of delay, return on investment, risk decision making, social cost, toxicity testing, value of information

## Abstract

A number of investigators have explored the use of value of information (VOI) analysis to evaluate alternative information collection procedures in diverse decision-making contexts. This paper presents an analytic framework for determining the value of toxicity information used in risk-based decision making. The framework is specifically designed to explore the trade-offs between cost, timeliness, and uncertainty reduction associated with different toxicity-testing methodologies. The use of the proposed framework is demonstrated by two illustrative applications which, although based on simplified assumptions, show the insights that can be obtained through the use of VOI analysis. Specifically, these results suggest that timeliness of information collection has a significant impact on estimates of the VOI of chemical toxicity tests, even in the presence of smaller reductions in uncertainty. The framework introduces the concept of the expected value of delayed sample information, as an extension to the usual expected value of sample information, to accommodate the reductions in value resulting from delayed decision making. Our analysis also suggests that lower cost and higher throughput testing also may be beneficial in terms of public health benefits by increasing the number of substances that can be evaluated within a given budget. When the relative value is expressed in terms of return-on-investment per testing strategy, the differences can be substantial.

## 1 | INTRODUCTION

Evidence-based risk assessment has become a cornerstone of public and population health risk decision making, integrating evidence on toxicity and exposure from multiple evidence streams. When the available evidence is insufficient to allow a decision to be made with confidence, consideration can be given to gathering additional evidence to strengthen the evidence base. The present paper focuses on the use of value of information (VOI) analysis to evaluate the utility of gathering additional evidence on the toxicity of chemicals. Specifically, we present a VOI analytic framework that builds on previous methodological work in this field, explicitly incorporating the value of additional test data resulting from reductions in the uncertainty in estimates of a chemical’s toxicity, the cost of delay in decision making that results from the time required for testing, and the expense of the testing. This work is motivated by the need to evaluate the large number of as yet untested chemicals that are present in the human environment, a challenge that may be addressed by employing more rapid and cost-effective toxicity-testing methodologies that have become available in recent years (Krewski et al., 2020; Thomas et al., 2019). Our VOI framework provides a basis for evaluating the trade-offs among the degree of uncertainty reduction provided by different tests for chemical toxicity and the duration and costs of those tests. Important questions pertaining to these trade-offs were posed in the U.S. National Academy of Sciences Report, *Science and Decisions: Advancing Risk Assessment* (National Research Council [NRC], 2009), but have not yet been systematically addressed. While not explored in this paper, the framework could also be used to evaluate value of the collection of additional information to reduce uncertainty in exposure.

VOI is a means of valuing the expected gain from reducing uncertainty by the collection of additional data. Determination of the value of the additional information begins with an evaluation of the implication of the new information for a risk-based decision making, which requires a model for the relationship of the uncertainty in the existing information and the cost of the uncertainty on the decision. VOI analysis determines the cost of errors in decisions that result from the uncertainty in existing information and how these costs will be reduced if additional information is obtained. The reduction in cost produced by additional information is the basis for assigning value to that information, which can then be compared to the cost of information collection. The cost, value, and timeliness of additional information can then be used to determine the net present value of the data collected. A systematic review by Keisler et al. (2014) documented applications of VOI in a wide range of disciplines, including agriculture, anthropology, chemistry, defense, ecology, economics, education, energy, environmental science, geology, information science, infrastructure, medicine, and transportation. Based on the collective experience accumulated to date with this methodology, VOI would appear to be a valuable tool for evaluating the benefits gained by collecting additional information to strengthen risk-based decision making in the presence of uncertainty.

Historically, most VOI applications in the health sciences prior to 1995 were based on uncertainty distributions that were either discrete or discretized (Yokota & Thompson, 2004). A classic example is provided by work of Lave et al. (1988), who evaluated the cost-effectiveness of lifetime rodent bioassays against no testing using EVSI calculated based on a 2 × 2 contingency table (positive/negative human carcinogenicity cross-classified with positive/negative lifetime rodent bioassays). The VOI analysis found that for most chemicals such tests are not cost-effective.

As VOI methodologies advanced, computational trends shifted toward simulation-based analysis. For example, Thompson and Evans (1997) investigated the value of collecting additional exposure, toxicological, and pharmacokinetic data for perchloroethylene, a solvent used in dry-cleaning. The decision context was more complex than the discrete VOI analyses mentioned above, with the affected population partitioned into subgroups based on potential exposure levels (workers, families of workers, customers, and the general public). Their results suggested that information on toxicity was possibly more valuable than information on exposure. VOI methods have also found application in determining the value of additional analytical measurements of soil contamination on risk mitigation decisions at hazardous waste sites (Back, 2007; Bates et al., 2014; Englehardt & Simon, 2000).

While VOI analysis is a highly useful approach to evaluating the benefits of collecting additional information to support risk decisions, it is by no means a simple undertaking. Andronis (2015) notes that valuation of consequences such as willingness-to-pay (WTP) for quality-adjusted-life-years (QALYs) gained, and value of a statistical life (VSL) must be chosen with care, as these choices can have a strong influence on the outcome of the analysis. Koffijberg, Knies et al. (2018) discusses additional factors that may affect both the outcome and the validity of the analysis, including, correlations among input parameters, uncertainty in model structure, and choice of time horizon and discount rate. Koffijberg, Rothery et al. (2018) further notes that external constraints such as the maximum allowable control cost and health equity considerations may alter the outcome of the analysis as well.

A key aspect of public health decision making with respect to potentially toxic chemicals present in the environment involves the choice between making an immediate decision with currently available information versus delaying a decision until additional data are collected and analyzed. This choice is often informed by the urgency of the public health need and the costs, in terms of both time and resources, of acquiring additional relevant information that may lead to decisions with less uncertainty. Given that the toxicological properties of only a small fraction of the more than 86,000 chemicals in the Toxic Substances Control Act (TSCA) inventory (United States Environmental Protection Agency [U.S. EPA], 2021) have been investigated in detail, it is important to evaluate the trade-offs between timeliness, cost, and the degree of uncertainty reduction that various testing strategies offer.

**FIGURE 1:**
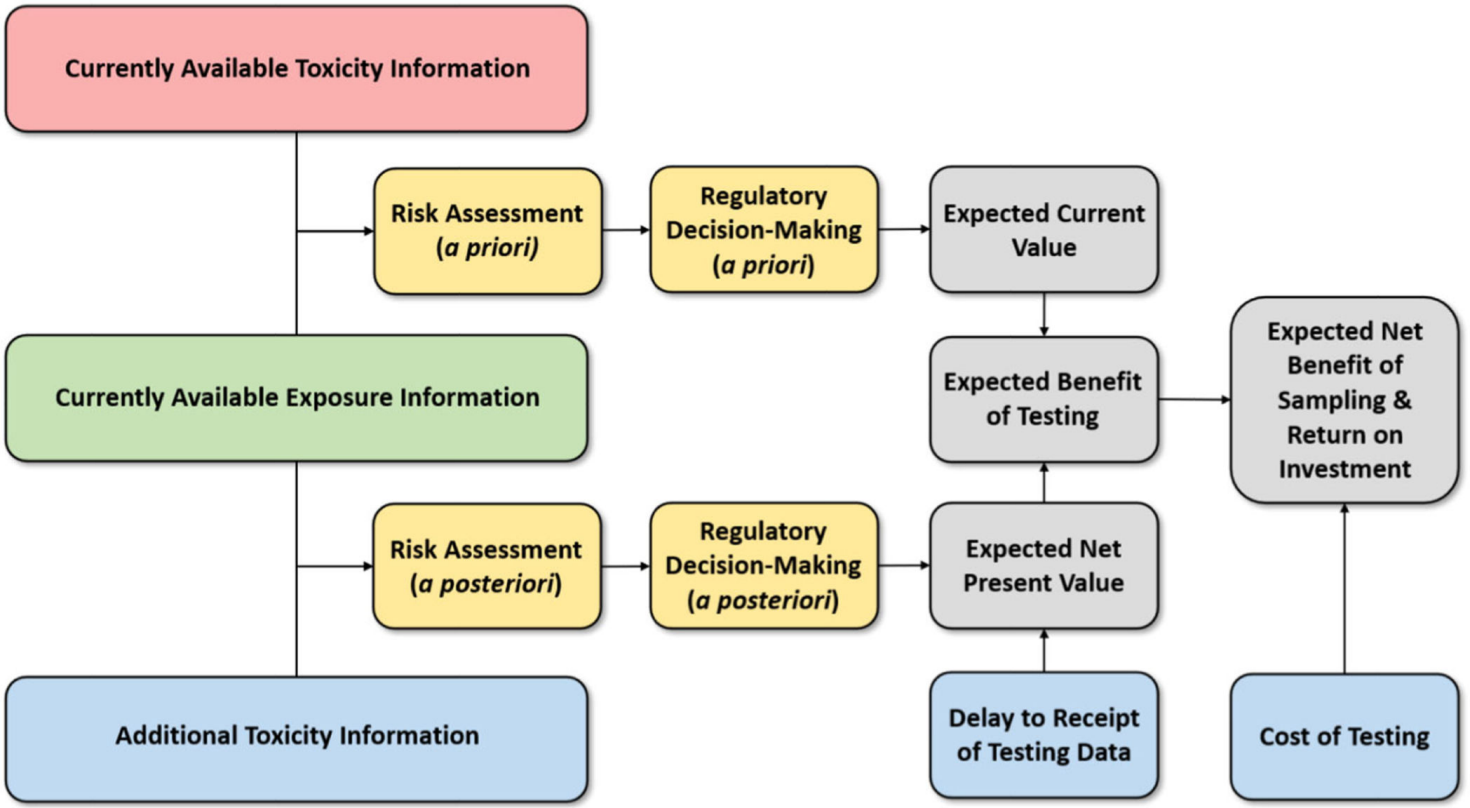
Steps and information flows in VOI analyses for the BRDM and TRDM. An *a priori* regulatory decision can be made by performing a risk assessment using currently available toxicity information and exposure information, with cost expressed in terms of expected current cost. An *a posteriori* regulatory decision can be made by incorporating the additional toxicity information with a corresponding delay in decision making. The value-of-information is calculated by comparing the expected cost associated with *a priori* and *a posteriori* regulatory decisions, incorporating the reduction in uncertainty, the delay in decision making, and the cost of collecting additional toxicity information

The goal of this paper is to present a framework for VOI analysis that can be used to compare different information collection techniques within the context of chemical toxicity testing, with an explicit consideration of the timeliness of the information. Our analytic framework for VOI analysis, outlined in schematic form in Figure 1, is described in detail in Section 2. Although an in-depth evaluation of the VOI associated with specific toxicity-testing methodologies is outside the scope of this paper, illustrative applications of how this can be done under hypothetical, but plausible, scenarios are provided in Section 3. A summary and discussion of our findings are given in Section 4, along with suggestions for further work in this area.

## 2 | METHODOLOGY

VOI analysis provides a methodology for valuing the results of additional toxicity-testing data in support of regulatory decision making. Valuation depends on how decisionmakers make their decisions to regulate or not regulate a chemical based on the available information on toxicity. In this paper, we consider two types of hypothetical decisionmakers, a benefit-risk decisionmaker (BRDM) who seeks to balance the cost of exposure mitigation and the resulting health benefits, and a target-risk decisionmaker (TRDM) whose objective is to control risks whenever the risk is thought to exceed a specified target risk level (TRL). Although neither decisionmaker perfectly emulates real-world regulatory decision making, both decision makers follow reasonable decision-analytic principles, with the primary difference being whether or not valuation of control cost is considered in the decision-making process.

In this section, we describe the steps involved in VOI analysis of different toxicity-testing methodologies. We begin by defining the models of risk that are used in the risk assessment steps (Figure 1, yellow boxes). These assessments are based on a set of parameters that reflect chemical toxicity and population exposure information (Section 2.1). In Section 2.2, we describe how uncertainty in toxicity and exposure parameters are characterized and how Bayesian updating of the parameter values is used to characterize the reduction in uncertainty in risk resulting from the collection of additional toxicity data. Objective functions that define value (Section 2.3) and decision rules (Section 2.4) for the BRDM and TRDM are then discussed. VOI metrics for valuing toxicity-testing data are presented in Section 2.5 (Figure 1, gray boxes).

### 2.1 | Population risk

In this paper, the average risk of a chemical within a given population, denoted here by R, is modeled as a function of its toxicity and exposure. Let x denote the level of exposure to the chemical of interest received by an individual from one or more sources and let θ denote the set of parameters that determine the risk level. Furthermore, let $f_{\exp}(x|\theta )$ be the probability density function of individual exposure within the population. The probability of an adverse effect to be present at a given exposure level x is given by

(1) $$G_{\text{tox}}(x|\theta )=P(\text{Adverse effect present}|X=x,\theta ).$$

To calculate population risk, let us suppose that θ can be partitioned into $\theta =\left[\theta_{\text{tox}},\theta_{\exp}\right]$, where $\theta_{\text{tox}}$ represents a set of parameters that govern the toxicity, and $\theta_{\text{exp}}$ control the exposure. Suppose further that $\theta_{\text{tox}}$ and $\theta_{\text{exp}}$ are statistically independent (reflecting independence of an individual’s exposure and tolerance), then the population average risk R can be expressed as

(2) $$R=R(\theta )=\int G_{\text{tox}}\left(x|\theta_{\text{tox}}\right)f_{\exp}\left(x|\theta_{\exp}\right)dx.$$

Following Chiu and Slob (2015) and Chiu et al. (2018), we assume that a lognormal distribution can be used to approximate both the inter-individual variation in susceptibility to the toxicity of the chemical as well as variation in exposures to the chemical from one or more sources. Since the lognormal (LN) distributions are described by their respective geometric means and geometric standard deviations, this assumption implies that $G\left(x|\theta_{\text{tox}}\right)$ represents the cumulative density function of $LN\left(\mu_{\text{tox}},\sigma_{\text{tox}}\right)$, and $f_{\exp}\left(x|\theta_{\exp}\right)$ denotes the probability density function of $LN\left(\mu_{\exp},\sigma_{\exp}\right)$, and thus $\theta =\left(\theta_{1},\theta_{2},\theta_{3},\theta_{4}\right)=\left(\mu_{\text{tox}},\sigma_{\text{tox}},\mu_{\exp},\sigma_{\exp}\right)$. As noted by Price et al. (2021), Equation (2) can be reexpressed as

(3) $$R=\text{Φ}\left(\frac{\mu_{\exp}-\mu_{\text{tox}}}{\sqrt{\sigma_{\text{exp}}^{2}+\sigma_{\text{tox}}^{2}}}\right),$$

where Φ(⋅) denotes the cumulative distribution function of the standard normal distribution.

Let k denote the proportionate reduction in mean exposure associated with a regulatory action. The resulting expected population risk, $R_{k}$, is then calculated as

(4) $$R_{k}=\int G_{\text{tox}}\left(x|\theta_{\text{tox}}\right)f_{\exp}\left(x|\theta_{\text{exp}}^{k}\right)dx,$$

where $\theta_{\exp}^{k}$ indicates that the mean exposure $\mu_{\exp}$ is reduced to $\mu_{\text{exp}}^{k}=\mu_{\exp}+\log_{10}(1-k/100)$.

These assumptions are generally reasonable and lead to tractable analytical solutions. Nonetheless, the proposed framework can be applied with other assumptions regarding the shape of the dose–response function, the distribution of variability in exposure, and the nature and extent of exposure reduction following risk mitigation action.

### 2.2 | Uncertainty, information collection, and Bayesian updating

Without the perfect information about the parameter set θ, the true population average risk cannot be obtained, and the lack of such information leads to uncertainty about the risk parameters that will be characterized in the form of an uncertainty distribution. Suppose that π(φ) denotes the prior uncertainty distribution based on currently available information about θ. Suppose further that additional information s∈𝓢 is collected to reduce uncertainty about θ. Following Bayes’ rule, the updated posterior uncertainty distribution κ(θ∣s) is given by

(5) $$\kappa (\theta |S=s)=\frac{L(s|\theta )\pi (\varphi )}{f(s)},$$

where L(s∣θ) is the likelihood function of S=s given θ, and f(s) is the unconditional probability density function of S at s.

Assume further that prior uncertainty distributions for each $\theta_{i}$ follows a lognormal distribution with parameters $\left(\mu_{\theta_{i}}^{0},u_{\theta_{i}}^{0}\right)$ for i=1,2,3,4. If the additional information S∣θ also follows a lognormal distribution with parameters $\left(\mu_{S},\sigma_{S}\right)$, then

(6) $$\kappa \left(\theta_{i}|S\right)\sim LN\left(\mu_{\theta_{i}}^{\prime },u^{\prime }\left(\theta_{i}\right)\right),$$

with

(7) $$\mu_{\theta_{i}}^{\prime }={\left(u_{\theta_{i}}^{\prime }\right)}^{2}\left(\frac{\mu_{\theta_{i}}^{0}}{{\left(u_{\theta_{i}}^{0}\right)}^{2}}+\frac{s}{\sigma_{S}^{2}}\right),$$

and

(8) $$u_{\theta_{i}}^{\prime }={\left(\frac{\sigma_{S}^{2}{\left(u_{\theta_{i}}^{0}\right)}^{2}}{\sigma_{S}^{2}+{\left(u_{\theta_{i}}^{0}\right)}^{2}}\right)}^{\frac{1}{2}}.$$

### 2.3 | Objective functions

In its most general form, VOI analysis seeks to optimize an objective function that depends on the decision context and decision criteria. In the VOI literature, the annualized social cost (ASC) has often been used as the objective function (see, for example, Taylor et al., 1993). Here, we use the total social cost (TSC) as the objective function. TSC builds on the ASC and better reflects the temporal aspects of risk decision making. The BRDM seeks to minimize the social cost by determining an optimal reduction in exposure (ORE). The TRDM, on the other hand, will reduce exposure when the population risk is considered high, with the corresponding reduction in health cost, valued in economic terms, effectively representing the objective function.

#### 2.3.1 | Annualized social cost

The ASC is the sum of the annual health cost and control cost. If a population is currently exposed to a chemical and receives a risk of an adverse effect, the social cost is the health cost of the adverse effects in the population. The control cost is the cost to reduce exposures incurred as a result of the decision that risk must be mitigated, with the health cost representing the cost the after the controls are applied. Let $ACC_{k}$ and $AHC_{k}$ denote the annual control cost and the annual health cost associated with reducing the exposure by k%, respectively. Then, at any given time in the future y, the ASC can be expressed as

(9) $${\text{ASC}}_{k}={\text{ACC}}_{k}+{\text{AHC}}_{k}=\frac{C_{k}}{{\left(1+r\right)}^{y-1}}+\frac{N_{y}VR_{k}}{{\left(1+r\right)}^{y-1}},$$

where, $N_{y}$ is the number of exposed persons at time $y,\;C_{k}$ is the annual cost of control due to the reduction in exposure by k%, $R_{k}$ is the residual risk of the adverse effect after the exposure is reduced by k%, V represents the cost of the specific health detriment being predicted, r is the discount rate used to determine the net present value of future benefits and costs.

#### 2.3.2 | Total social cost

In practice, it is important to consider the time required to arrive at a decision and implement actions based on it, rather than simply comparing the impact of uncertainty reduction on health cost (HC) and control cost (CC) at a given time point, which may not adequately represent the health cost incurred during the implementation period. Recognizing this temporal dimension of risk-based decision making, the proposed framework uses the TSC rather than the ASC. The TSC is defined as the summation of the net present value of all costs over the time horizon of interest. Suppose that there are J toxicity tests (with j=0 corresponding to no testing). For each test method j=0,…,J and risk mitigation action resulting in a proportionate reduction in exposure of 0≤k≤100%, we need the following information: the time required to conduct *j*th test $\left(t_{\text{test},j}\right)$; the time required to evaluate the results based the *j*th test and make a decision $\left(t_{\text{DM},j}\right)$; and the time required to implement measures to reduce exposure by $k\%\left(t_{\text{imp},k}\right)$.

Then, over a predetermined time horizon $y_{\text{TH}}$, the total control cost (TCC) and the reduction in total health cost (THC) are incurred once the regulatory action is implemented (i.e., $y_{\text{imp},j,k}=t_{\text{test},j}+t_{\text{DM},j}+t_{\text{imp},k}$).


(10) $${\text{TSC}}_{k}^{j}={\text{TCC}}_{k}^{j}+{\text{THC}}_{k}^{j}\;=\sum_{y=y_{\text{imp},j,k}}^{y_{TH}}\frac{C_{k}}{{\left(1+r\right)}^{y-1}}+\left[\sum_{y=1}^{y_{TH}}\frac{N_{y}B_{y}RV}{{\left(1+r\right)}^{y-1}}-\sum_{y_{\text{imp},j,k}}^{y_{TH}}\frac{N_{y}B_{y}\left(R-R_{k}\right)V}{{\left(1+r\right)}^{y-1}}\right].$$

Here, the superscript *j* denotes the delay in decision making associated with the time taken to collect and evaluate data for *j*th toxicity test; the subscript *k* denotes the proportion of reduction in exposure; and y_imp,*j*,*k*_ is the time to implement the decision based on the *j*th toxicity testing to reduce exposure by *k*%, y_*TH*_ is the time horizon, *R* is the risk of the adverse effect due to exposure without control strategy, *B_y_* is the risk annualization factor to convert *R* (e.g., from lifetime risk to annual risk).

### 2.4 | Decision rules

A key analytical component of VOI analysis is the characterization of the decisionmaker and their changing behavior in response to new information. This section presents the objective decision rules for the TRDM and the BRDM.

The TRDM’s risk management goal is to control exposure to the substance of interest if the risk is found to be above the TRL. Thus, if we know the true risk $R_{\text{true}}$, the decision rule D(R) for the TRDM can be specified as

(11) $$D_{\text{TRL}}\left(R_{\text{true}}\right)=\{\begin{array}{ll} 1 & \text{if}\;R_{\text{true}}>\text{TRL}\\ 0 & \text{if}\;R_{\text{true}}\leq \text{TRL} \end{array}.$$

If, for example, the TRDM were to reduce the mean exposure by 90% when the decision is to implement an exposure mitigation measure (corresponding to a reduction of 1 unit on the ${\text{log}}_{10}$ scale), the corresponding action A(R) would be to shift the mean of the lognormal exposure distribution, which can be specified as:

(12) $$A_{\text{TRL}}(R)=\{\begin{array}{l} \mu_{\exp}\to {\mu^{\prime }}_{\exp}∋{\mu^{\prime }}_{\exp}=\mu_{\exp}-1\;\text{if}\;D_{\text{TRL}}(R)=1\\ \mu_{\exp}\to \mu_{\exp}\;\text{otherwise}. \end{array}$$

In practice, the TRDM would likely consider additional factors such as the level of risk and variability in risk among population subgroups, available control technologies, and exposure pathways in determining an appropriate exposure mitigation strategy, rather than invoking a fixed proportionate reduction in exposure as in this simplified action.

**FIGURE 2:**
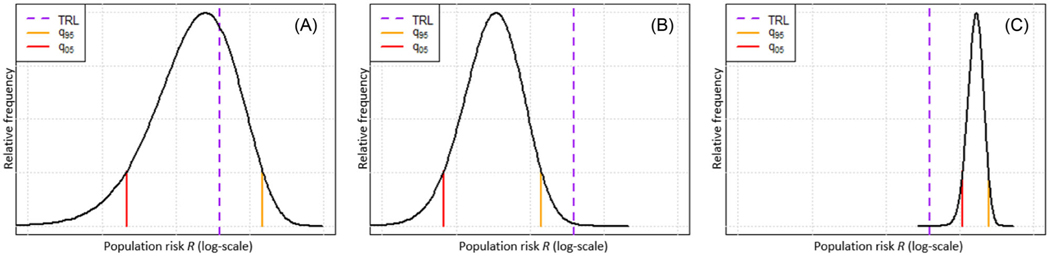
Illustration of TRDM decision making under prior and posterior uncertainty. (A. Prior uncertainty distribution about risk, where the TRL is greater than $q_{05}$ but less than $q_{95}$ and hence no decision can be made; B. Posterior uncertainty distribution about risk given sample information with a reduced uncertainty range. Since the TRL is greater than $q_{95}$, the TRDM would conclude that no regulation is required; C. Posterior uncertainty distribution about risk given sample information with a further reduced uncertainty range. As $q_{05}$ is greater than the TRL, the TRDM would take exposure mitigation action).

Under the presence of uncertainty about R, the TRDM requires assurance that the risk is above or below the TRL before concluding whether risk mitigation is, or is not, required (Figure 2). If the 5th ($q_{05}$) and 95th ($q_{95}$) percentiles (other percentiles could also be used) of the uncertainty distribution of the average population risk includes the TRL, the TRDM would want additional data in order to decide whether risk reduction action is required (Figure 2A). If the prior uncertainty is such that the TRDM is unable to make a decision ($q_{05}$ ≤ TRL ≤ $q_{95}$), the TRDM requires additional toxicity-testing data. If the 95th percentile of the posterior uncertainty distribution of the population risk is below the TRL, then the TRDM would not need to take exposure mitigation action (Figure 2B). Conversely, if the 5th percentile of the posterior distribution was above the TRL, the TRDM would take action to reduce risk (Figure 2C). Mathematically, the decision rule for the TRDM with uncertainty can be expressed as

(13) $$D_{\text{TRL}}(R)=\{\begin{array}{ll} 1 & \text{if}\;q_{05}>\text{TRL}\\ 0 & \text{if}\;q_{95}\leq \text{TRL}\\ -1 & \text{otherwise} \end{array},$$

where $D_{\text{TRL}}=1,0,-1$ denotes a case in which risk reduction action is required, not required, or unclear, respectively, with the corresponding action A(R) given in Equation (12).

In contrast, the BRDM would engage in exposure mitigation if the economic value of the public health benefits of exposure mitigation is greater than the control cost. This decision rule is specified by:

(14) $$D\left(R_{\text{true}}\right)=\{\begin{array}{ll} 1 & \text{if}\;\exists \;R_{\text{true}}^{\prime }<R_{\text{true}}∋\text{TSC}\left(R_{\text{true}}^{\prime }\right)<\text{TSC}\left(R_{\text{true}}\right)\\ 0 & \text{otherwise} \end{array},$$

with corresponding action:

(15) $$A\left(R_{\text{true}}\right)=\{\begin{array}{l} R_{\text{true}}\to R_{\text{true}}^{*}∋R_{\text{true}}^{*}=\text{argmin TSC}\left(R_{\text{true}}\right)\;\text{if}\;D\left(R_{\text{true}}\right)=1\\ R_{\text{true}}\to R_{\text{true}}\text{if}\;D\left(R_{\text{true}}\right)=0. \end{array}$$

Here, $R_{\text{true}}^{*}$ denotes the reduced risk level corresponding to the minimum TSC value (denoted by the “argmin” operator).

**FIGURE 3:**
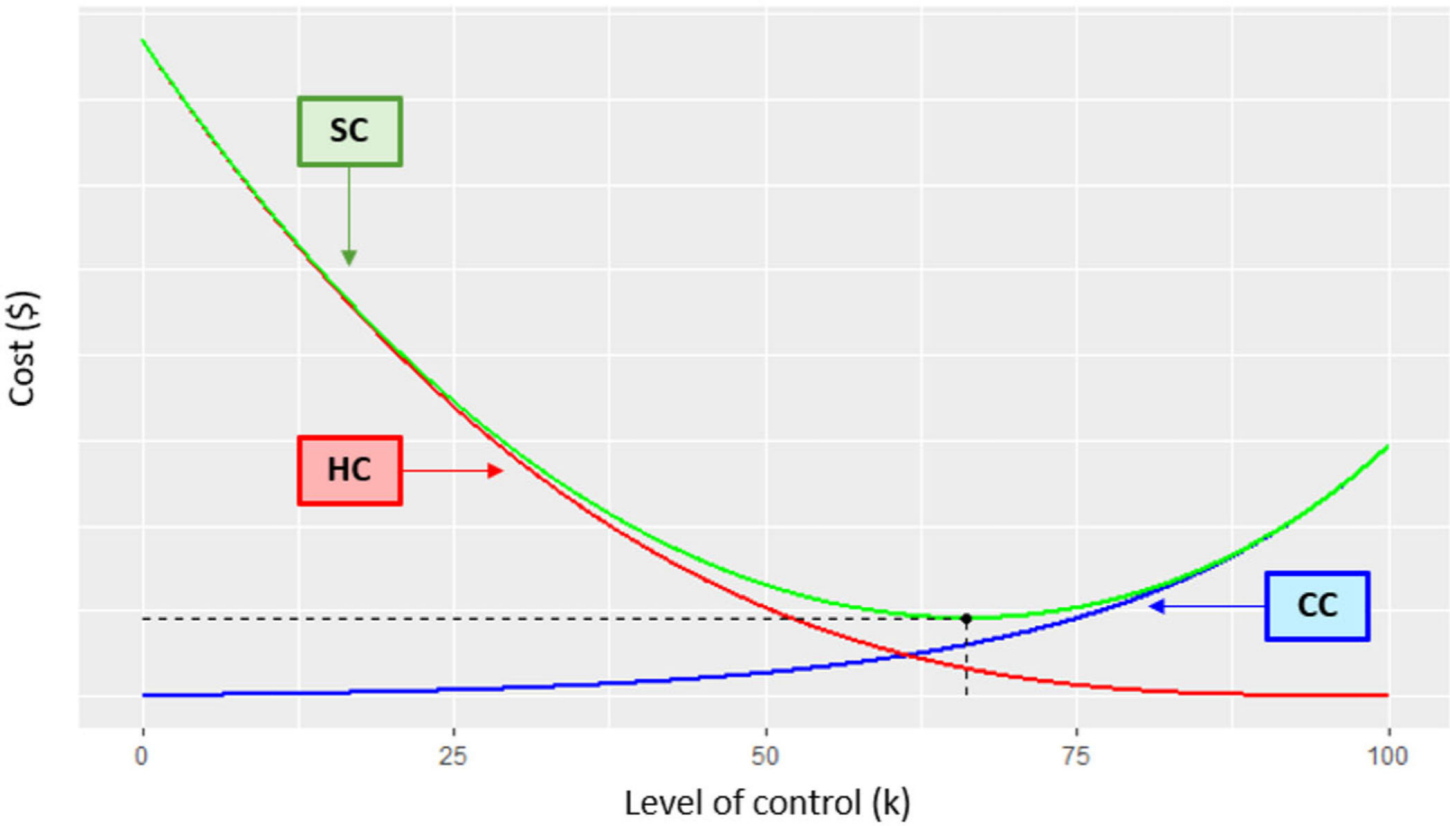
Illustration of social cost (SC) as a function of increasing control cost (CC) and decreasing health cost (HC). (Optimal reduction in exposure that minimizes social cost is denoted by the solid circle)

The decision-making process for BRDM is illustrated graphically in Figure 3. As the level of control increases, the health cost correspondingly decreases. The TSC achieves a minimum by striking a balance between the benefits of reducing the health cost and the cost of increasingly stringent control. The ORE corresponds to the point where the TSC is minimized. In the presence of uncertainty, the BRDM would use the expected risk E(R) instead of the true risk $R_{\text{true}}$ and expected total social cost (ETSC) defined below, instead of the TSC, in Equations (14) and (15). Note that although the BRDM is always able to make a decision on the level of exposure mitigation required due to the balancing of benefits and risk included in the TSC, the TRDM will not be able to make a decision in cases where the level of uncertainty precludes a determination of whether or not population risk is above or below the TRL.

### 2.5 | Measuring value of information

As noted previously, the BRDM is interested in minimizing the TSC across the uncertainty distribution as the basis for evaluating the VOI of toxicity-testing data and determining the ORE. The TRDM undertakes a similar analysis by evaluating the reduction in (expected) THC when a decision is made to reduce exposure. Within the context of the framework used in the present paper, the value of reducing uncertainty can be gauged using appropriate metrics comparing prior and posterior uncertainties using Bayesian updating after additional data collection. Details of the VOI analyses conducted by the BRDM and TRDM are presented below.

#### 2.5.1 | Expected total social cost

In the presence of uncertainty, the true risk is unknown. Thus, given an uncertainty distribution on R, based on prior information, $h^{0}(R)$, the ETSC with k% exposure mitigation is given by

(16) $${\text{ETSC}}_{k}^{0}(R)=E\left({\text{TSC}}_{k}^{0}(R)\right)=\int {\text{TSC}}_{k}^{0}(R)h^{0}(R)dR.$$

#### 2.5.2 | Expected total health cost

For the TRDM, the expected total health cost (ETHC) based on prior information is given by:

(17) $${\text{ETHC}}_{k=90}^{0}(R)={\text{I}}_{\left[D_{\text{TRL}}(R)=1\right]}\int {\text{THC}}_{k=90}^{0}(R)h^{0}(R)dR\;\;+{\text{I}}_{\left[D_{\text{TRL}}(R)\neq 1\right]}\int {\text{THC}}_{k=0}^{0}(R)h^{0}(R)dR,$$

where I[⋅] denotes an indicator function for the condition (⋅). Note that the first term on the right-hand side of Equation (17) reflects the case in which TRL < *q*_05_ and exposure is reduced by 90%, and the second term corresponds to the case where TRL > *q*_05_ which results in no regulatory action taken.

#### 2.5.3 | Expected value given current information

Since the BRDM is interested in minimizing the ETSC, the ORE based on the prior decision $k^{0}$ is

(18) $$k^{0}=\underset{k}{\text{argmin}}\;{\text{ETSC}}_{k}^{0}(R).$$

The corresponding ETSC, called the expected value given current information (EV|CI), is given by

(19) $$\text{EV}|\text{CI}={\text{ETSC}}_{k^{0}}^{0}(R),$$

where EV|CI represents the minimum ETSC using currently available information only.

For the TRDM, VOI analysis is useful when the prior uncertainty is sufficiently great to preclude making a decision. In this case, the corresponding EV|CI can be obtained as

(20) $$\text{EV}|\text{CI}={\text{ETHC}}_{k=0}^{0}(R),$$

where k=0 corresponds to the ETHC associated with current unmitigated exposures.

#### 2.5.4 | Expected value of immediate perfect information

To calculate the expected value of immediate perfect information (EVIPI), consider first the BRDM. Let $k^{0*}=k^{0*}|R=\underset{k}{\text{argmin}}\;{\text{TSC}}_{k}^{0}(R)$, that is, $k^{0*}$ is the locally optimal action for a given risk R. The expected value given immediate perfect information (EV|IPI) is then defined as the TSC under locally optimal action, integrated over the prior uncertainty distribution of R

(21) $$\text{EV}|\text{IPI}=\int {\text{TSC}}_{k^{0*}}^{0}(R)h^{0}(R)dR.$$

(Note that even with immediate perfect information, integration in Equation (21) is still required because the [preposterior] valuation is done before the hypothetical instantaneous toxicity test is conducted.) As EV|IPI denotes the minimal ETSC associated with making an optimal decision based on the prior uncertainty distribution, the EVIPI—the expected value of always making an optimal decision, can be expressed as

(22) EVIPI=EV|CI−EV|IPI,

For the TRDM, EVIPI is also calculated as in Equation (22), but with:

(23) $$\text{EV}|\text{IPI}=\int [{\text{THC}}_{k=90}^{0}(R){\text{I}}_{\left[D_{\text{TRL}}(R)=1\right]}\;\;+{\text{THC}}_{k=0}^{0}(R){\text{I}}_{\left[D_{\text{TRL}}(R)\neq 1\right]}]h^{0}(R)dR.$$

In practice, it is impossible to remove uncertainty completely, and hence the EVIPI is only of theoretical interest. It can, however, be used as an upper limit on the cost of collecting of further information, over and above the value of information obtained via the *j*th toxicity test.

#### 2.5.5 | Expected value of immediate partial perfect information

Since toxicity testing can only reduce uncertainty about toxicity parameters, the uncertainty about exposure parameters will be unchanged even when perfect information about toxicity parameters is obtained. When perfect information is obtained only for a subset of parameters, ϑ∈θ, then such information is called partial perfect information. For the BRDM, the expected value given immediate partial perfect information (EV|IPPI) is then defined as

(24) $$\text{EV}|\text{IPPI}=\int \left[\min\int {\text{TSC}}_{k}^{0}(R|\vartheta )h(R|\vartheta )dR\right]h^{0}(\vartheta )d\vartheta ,$$

where h(R∣ϑ) denotes the uncertainty distribution about risk given a partial perfect information. Thus, the expected value of immediate partial perfect information (EVIPPI) is given by:

(25) EVIPPI=EV|CI-EV|IPPI.

For the TRDM, EVIPPI is also determined from Equation (25), but with

(26) $$\text{EV}|\text{IPPI}=\int \left[{\text{ETHC}}_{k}^{0}(R|\vartheta )\right]h(\vartheta )d\vartheta .$$

#### 2.5.6 | Expected value of delayed sample information and the cost of delay

Although it is impossible to completely eliminate uncertainty, it is possible to reduce uncertainty by gathering additional information. Using the *j*th toxicity test, if sample information $s_{j}\in S_{j}$ is realized, such information can be used to update the uncertainty distribution for the population risk R. In practice, the collection of additional information requires time, which translates into a delay in decision making. Incorporating such a delay, we can obtain the expected value given delayed sample information (EV|DSI) for the BRDM using the *j*th toxicity test as

(27) $$\text{EV}|{\text{DSI}}^{j}=\int \left\{\min\left[{\text{ETSC}}_{k}^{j}\left(R|s_{j}\right)\right]\right\}f\left(s_{j}\right)ds_{j},$$

with the associated EVDSI

(28) $${\text{EVDSI}}^{j}=\text{EV}|\text{CI}-\text{EV}|{\text{DSI}}^{j}.$$

Suppose now that such information can be obtained without delay. Then the expected value given immediate sample information (EV|ISI), and corresponding expected value of immediate sample information (EVISI), can be calculated by replacing ${\text{TSC}}_{k}^{j}(R)$ with ${\text{TSC}}_{k}^{0}(R)$ in Equation (27), and

(29) $${\text{EVISI}}^{j}=\text{EV}|\text{CI}-\text{EV}|{\text{ISI}}^{j},$$

respectively.

Similarly, for TRDM, EV|DSI using the *j*th toxicity test is defined as

(30) $$\text{EV}|{\text{DSI}}^{j}=\int {\text{ETHC}}_{k}^{j}\left(R|s_{j}\right)f\left(s_{j}\right)ds_{j},$$

with corresponding EVDSI^*j*^ derived using Equation (28). Furthermore, ${\text{EV|ISI}}^{j}$ is derived by substituting ${\text{ETHC}}_{k}^{0}\left(R|s_{j}\right)$ for ${\text{ETHC}}_{k}^{j}\left(R|s_{j}\right)$ in Equation (30) and ${\text{EVISI}}^{j}$ using Equation (29).

Since the quality of information is the same for both ${\text{EVDSI}}^{j}$ and ${\text{EVISI}}^{j}$, the difference between these two values can be attributed to the delay in the decision making. Thus, the cost of delay (CoD) can be defined as

(31) $${\text{CoD}}^{j}={\text{EVISI}}^{j}-{\text{EVDSI}}^{j}.$$

Hence, the EV|CI can be partitioned to

(32) $$\text{EV}|\text{CI}=\text{EV}|{\text{ISI}}^{j}+{\text{CoD}}^{j}+{\text{EVDSI}}^{j}.$$

The concept of delayed information, and the cost associated with that delay, is also relevant when evaluating both perfect information and partial perfect information. The cost of delay given perfect information is defined by

(33) $${\text{CoD}}^{\text{PI}}=\text{EVIPI}-\text{EVDPI},$$

where EVDPI denotes the expected value of delayed perfect information calculated from Equation (22) by incorporating the delay involved in the calculation of TSC.

Similarly, the cost of delay given partial perfect information is defined by

(34) $${\text{CoD}}^{\text{PPI}}=\text{EVIPPI}-\text{EVDPPI},$$

where EVDPPI denotes the expected value of delayed partial perfect information calculated from Equation (25), including the delay involved in the calculation of TSC.

#### 2.5.7 | Expected net benefit of sampling

Although the EVDSI reflects the potential benefit of obtaining additional toxicity test data, it does not consider the cost of testing (CoT), representing the direct cost of collecting additional information. The decisionmaker may calculate the ENBS as

(35) $${\text{ENBS}}^{j}={\text{EVDSI}}^{j}-{\text{CoT}}^{j}.$$

A positive ENBS implies that ${\text{CoD}}^{j}$ and ${\text{CoT}}^{j}$ are smaller than the value of the additional information provided by the *j*th toxicity test. Whereas negative ENBS values mean that the additional information collected does not provide sufficient value to offset the ${\text{CoD}}^{j}$ and ${\text{CoT}}^{j}$. In practice, the ENBS represents an attractive metric for gauging VOI since it also takes into account the costs of testing and delay.

#### 2.5.8 | Return on investment

Another useful measure the expected benefit of collecting additional toxicity data is the ROI, defined as

(36) $${\text{ROI}}^{j}=\frac{{\text{ENBS}}^{j}}{{\text{CoT}}^{j}}.$$

Similar to ENBS, a positive ROI value indicates that the toxicity test is beneficial, while a negative ROI implies that the testing is not worthwhile. It may be noted that, unlike the ENBS metric that is measured in monetary units, the ROI metric is the value per dollar spent ($/$) and is a unitless ratio. Based on the ROI, the best toxicity-testing procedure j* is determined as

(37) $$j^{*}=\underset{j}{\text{argmax}}\;{\text{ROI}}^{j}.$$

Like the ENBS, the ROI also represents an important metric in practical applications since it also considers the cost of testing.

## 3 | ILLUSTRATIVE APPLICATIONS

In order to demonstrate how VOI is calculated, we present two illustrative applications—one with a fatal outcome and another an acute health outcome—designed to represent situations that could be of general interest to decisionmakers. Each application consists of three scenarios defined by the parameters given in Table 1, with varying prior uncertainty. These examples address aspects of VOI analyses relevant to the selection of toxicity-testing methodologies, the value of testing results, the cost of testing, the impacts of duration of testing, and the impacts of different levels of uncertainty in the estimates of toxicity.

In these scenarios, we set the size of the target population to be N=350M people, roughly the size of the U.S. population. TH denotes the time horizon over which the VOI calculation is done, and the time required to implement a regulation designed to reduce population health risk is denoted by $t_{\text{imp}}$. In all of the scenarios considered here, we set TH=20 years, allowing a sufficient time to capture the costs and benefits associated with the risk reduction strategy selected by the decisionmaker. We set $t_{\text{imp}}$ to be 2 years, reflecting notional delays in implementation of exposure mitigation and realization of the health benefits.

To illustrate the method, we compare the VOI associated with two toxicity-testing strategies—denoted Test A and Test B—that provide toxicity information that can be used to estimate population health risk. The times required to conduct each of these testing strategies and evaluate the results are denoted by $t_{A}$ and $t_{B}$, respectively. Whereas the results from Test A are available to decisionmakers within a year $\left(t_{A}=1\right)$, the more intensive Test B requires 5 years to complete $\left(t_{B}=5\right)$. Test A reduces the prior uncertainty about $\mu_{\text{tox}}$ to within four orders of magnitude (OM) on the log scale (corresponding to 99.975% of the range of uncertainty distribution, spanning $7.325u^{A}\left(\mu_{\text{tox}}\right);u^{A}\left(\mu_{\text{tox}}\right)=4/7.325=0.55$), and that the more demanding Test B reduces uncertainty to within 2 OM (also corresponding to 99.975% of the reduced range of uncertainty; $u^{B}\left(\mu_{\text{tox}}\right)=0.27$) of the true value of $\mu_{\text{tox}}$. In the scenarios presented in this section, Test A represents a more timely, lower cost $\left({\text{CoT}}_{A}=\$5\text{K}\right)$ experimental approach as compared to the slower, more expensive Test B $\left({\text{CoT}}_{B}=\$5\text{M}\right)$. Note that Test A and Test B effectively trade-off cost and timeliness against uncertainty reduction, with the slower and more expensive Test B yielding a greater reduction in uncertainty.

To explore the effect of varying level of prior uncertainty, we consider three scenarios. In scenario 1, we assume that the prior uncertainty only exists about $\mu_{\text{tox}}$, with a corresponding uncertainty range of $7\;\text{OM}\left(u^{0}\left(\mu_{\text{tox}}\right)=0.96\right)$, representing a case where there is little information available about toxicity. (This assumption is motivated by results from Krewski et al. (1993a) and Chiu et al. (2018), based on empirical results on the variation in carcinogenic potency of chemicals and points of departure^1^ for subchronic and chronic noncancer endpoints, respectively.) In scenario 2, the prior uncertainty about $\mu_{\text{tox}}$ is assumed to be $5\;\text{OM}\left(u^{0}\left(\mu_{\text{tox}}\right)=0.68\right)$, representing the case where some preliminary information is available. The third scenario introduces uncertainty about $\mu_{\exp}$, splitting the 7 OM in combined prior uncertainty about parameters $\mu_{\text{tox}}$ and $\mu_{\exp}$ into 5 OM and $\sqrt{24}=\sqrt{7^{2}-5^{2}}\;\text{OM}$ (recall that $u{\left(\mu_{\exp}-\mu_{\text{tox}}\right)}^{2}=u{\left(\mu_{\exp}\right)}^{2}+u{\left(\mu_{\text{tox}}\right)}^{2}$, respectively. Comparison of scenarios 1 and 2 allows a study of the effect of the magnitude of prior uncertainty about $\mu_{\text{tox}}$, while comparison between scenarios 1 and 3 provides an insight to the effect of unreducible uncertainty.

A discount rate of r=5% will be used to adjust all future costs and benefits to their net present values. The use of a 5% discount rate is based on recommendations from the U.S. EPA Science Advisory Board (SAB) as a central rate between 3% and 7%, which represent the estimated consumption rate of interest and opportunity cost of capital, respectively (U S. EPA, 2004). The maximum annualized control cost associated with complete elimination of exposure is set to ${\text{ACC}}_{\max}=\$2.2\;\text{B}$ as the default value. For context, this value represents the average cost of controlling air pollutants such as acid gas, ${\text{PM}}_{2.5}$, and ${\text{SO}}_{2}$, annually for each of these three pollutants (U.S. EPA, 2011). The annualized fixed control cost is denoted by ${\text{ACC}}_{\text{F}}$, which, for simplicity, is set to 0. Using the following nonlinear function defined between ${\text{ACC}}_{F}$ and ${\text{ACC}}_{\text{max}}$ (other monotonically increasing functions could also be employed), the control cost can be calculated as

(38) $${\text{ACC}}_{k}={\text{ACC}}_{\text{F}}+\left({\text{ACC}}_{\max}-{\text{ACC}}_{\text{F}}\right)\frac{10^{\eta k}-1}{10^{\eta }-1},$$

where η>0 controls the steepness of the cost function. The default value of 𝜂 is set to 2 (with other values considered in sensitivity analyses).

The two applications of the framework also consider the decision rules for the TRDM and BRDM. An important difference between the two sets of decision rules relates to the level of the exposure reduction chosen by these two decisionmakers. As described above, the BRDM selects the level of exposure reduction to minimize the ETSC and the TRDM reduces the geometric mean of exposure by 90% when $q_{05}$ exceeds the TRL. (Although other risk reduction actions could be contemplated, we chose a fixed proportionate reduction in risk for simplicity.)

In the first illustrative application focusing on mortality, we used $8.8M^2^ as the value of a statistical life (VSL) to represent the economic benefit of risk reduction. The toxicity and exposure parameters for a hypothetical chemical of interest are given in Table 1. In this application, we used an ${\text{ED}}_{50}$ value of 50 mg/kg-bw/day, which is close to the center of the distribution of carcinogenic potency given by Krewski et al. (1993b, Figure 1). We further assume that a chemical with toxicity equal to the median of toxicity distribution presents a lifetime risk of 10^−8^, reflecting a situation in which population risk associated with a chemical exposure is very low. For the fatal outcome, the TRL is set to 10^−7^ over the course of lifetime. Specification of the ${\text{ED}}_{50}$ and population median risk imply a population median exposure of 7.7 × 10^−4^ mg/kg-bw/day. We further assume that inter-individual susceptibility varies in accordance with a lognormal distribution with a log GSD of 0.697, and that variation in exposure also follows a lognormal distribution with a log GSD of 0.5. The log GSD of 0.697 assumed for interindividual variation in susceptibility is taken from the upper end of the range of values suggested by World Health Organization and International Program on Chemical Safety (WHO & IPCS, 2017) for the uncertainty for intraspecies variability in hazard characterization. To convert the lifetime risk to annualized risk, we use $B_{y}=1/80$, based on an assumed life expectancy of 80 years.

In the second illustrative application, which focuses on an acute health effect, we consider a restricted airway event valued at $50 per occurrence (U.S. EPA, 2012). For this outcome, we assume the median daily risk is $R=10^{-6}$, with a (daily) TRL of 10^−5^. The annualization factor of $B_{y}=365$ is used to convert the daily risk to annualized risk.

### 3.1 | VOI analyses

#### 3.1.1 | Target-risk decision making

Table 2 presents the relevant VOI metrics for the TRDM for the fatal outcome. In Scenario 1, EV|CI ($44, 784M) is quite high, reflecting insufficient evidence to regulate chemicals that pose a risk greater than the TRL. With perfect information, the ETHC would be reduced by $36, 819M from controlling exposure for any chemical that exceeds the TRL. While the value of testing information without delay is greater for $\text{Test B}\;\left({\text{EVISI}}_{\text{B}}=\$36,704\text{M}\right)$ than $\text{Test A}\;({\text{EVISI}}_{\text{A}}=\$31,414\text{M})$, the EVDSI ($28, 855M) for Test A is greater than that of Test B ($23, 110M) due to the greater cost of delay in realizing results with Test B. Taking the cost of testing into account, the ROI for Test A is 5, 770, 902, which is more than 1200 times greater than that of Test B (4621).

Depending on the informativeness of the available toxicity-testing data, the TRDM may or may not be able to make a decision as to the need for risk reduction. As shown in Table 3 there is a 35.4% chance in Scenario 1 that perfect information would identify an untested chemical as requiring regulation, and a 64.6% that perfect information would show that the chemical would require no regulation. Test A provides sufficient evidence to conclude whether a chemical requires regulation in 5.7% of the cases, while Test B provides sufficient information in 18.8% of the time. Despite the greater ability of Test B to enable decisions, both tests demonstrate similar EVISI results. This can be attributed to the fact that Test A will lead to a 90% reduction in exposure in cases where the population risk is high. In Scenarios 2 and 3, there is a substantially higher likelihood of making a decision to regulate a chemical with Test B than Test A (Table 3), with notably greater EVDSI and ENBS values for Test B. However, given the 1,000-fold difference in the cost of testing, the ROI for Test A is still favorable compared to Test B (Table 2). Similar results are observed with the acute outcome.

#### 3.1.2 | Benefit-risk decision making

Table 4 presents the results of our VOI analyses for the BRDM. In scenario 1, where the prior uncertainty about $\mu_{\text{tox}}$ spans 7 OM, the ORE that minimizes the ETSC is $k^{0}=73$, with an EV|CI of $19, 510M. If we have perfect information about $\mu_{\text{tox}}$ without delay, the ETSC would be reduced by $11, 618M, accounting for 59.5% of the EV|CI. In this scenario, the EVISI for Test A ($2, 253M) and for Test B ($2, 464M) are close to the EVIPI, indicating that the information provided by these two tests, if available without delay, results in a near-optimal VOI. However, the CoD for Test B, reflecting the 5-year period before the results are available for decision making, is $2, 841M, whereas the CoD for Test A, which only takes one year to complete, is only $654M. The CoD needs to be subtracted from EVISI to obtain the EVDSI.

When the delay in testing is taken into account (e.g., EV|CI minus EVDSI), Test A reduced the ETSC by $6, 958M, and Test B increased ETSC by $2, 263M. This increase for Test B is due to the larger health costs resulting from the 5 years of delay in data availability and subsequent regulatory controls. The positive EVDSI value for Test A indicates that the reduction in uncertainty with Test A is beneficial even with a 1-year delay in decision making, whereas this is not the case with Test B. Because of the lower cost of testing, Test A demonstrates a ROI of 1, 391, 550 (the value of information achieved per unit cost), than Test B, which yields a negative ROI (−454) due to the appreciable cost of delay.

In Scenario 2, the prior uncertainty about $\mu_{\text{tox}}$ is 100-fold smaller than Scenario 1. Scenario 2 produced a smaller prior ORE (24%), which in turn led to a significantly smaller CoD. This results in a reduced ROI value for Test A (129, 140), while ROI for Test B becomes positive (139). Unlike Scenarios 1 and 2, Scenario 3 acknowledges uncertainty in population mean exposure. Consequently, the reduction in uncertainty about the population average risk R following toxicity testing is smaller in Scenario 3. While the EVISI values have been drastically reduced, the CoD values that similar to those in Scenario 1 lead to the EVDSI, ENBS, and ROI to be all smaller in Scenario 3 than Scenario 1.

Although the per incident health cost is much smaller for the acute outcome than the fatal outcome, the higher risk and the possibility of the occurrence of multiple incidents leads to greater EV|CI values. In all three scenarios considered, Test A produced greater EVDSI, ENBS, and ROI values.

Further illustrative applications of our VOI methodology (results not shown) indicate that when the population average risk is small and the risk mitigation costs are high, the optimal reduction in exposure for both Tests A and B can be zero. This implies that when the population risk is low, both Tests A and B reduce the prior uncertainty to the point where the BRDM will be able to easily decide that any additional risk reduction is unjustified. Since the cost of Test A is 1,000-fold less than that of Test B and occurs sooner, Test A would be preferred in such cases. Similarly, when the population average risk is very high and control costs are low, both tests easily confirm that aggressive risk mitigation is required. Since both tests reach the same conclusion regarding the need for risk mitigation and Test A has a lower cost and a greater timeliness than Test B, Test A is again preferred. In intermediate cases, where the risk is neither extremely low nor extremely high, some degree of exposure mitigation will be required to minimize the ETSC: Test B is more likely to allow the BRDM to make an optimal decision, implying that the greater uncertainty reduction may be more compelling than the timeliness of information. It is these cases where a formal VOI analysis, including the key metrics ENBS and ROI, will be of most value in guiding the BRDM in making optimal risk reduction decisions that results in the greatest reduction in ETSC. A similar pattern is reported to occur in an analysis of testing methodologies performed using cost-effectiveness analysis (Price et al., 2021).

In the illustrative applications as discussed above, the BRDM takes a decision to regulate more often than the TRDM, who is able to take a decision to regulate or not a regulate in the presence high posterior uncertainty (Table III).

### 3.2 | Trade-offs between uncertainty reduction and timeliness

Both the timeliness of information collection and the associated reduction in uncertainty are critical determinants of the overall effectiveness of different toxicity-testing strategies. Tables S1 and S2 in the Supporting Information show the change in EVDSI for the TRDM as a function of testing time (ranging from 0 to 10 years with a yearly increment), as well as the posterior uncertainty in $\mu_{\text{tox}}$ (ranging from 0% to 100% reduction in prior uncertainty about $\mu_{\text{tox}}$, with 100% reduction reflecting perfect toxicity information), for the illustrative applications discussed above. While the specific relationship between timeliness and uncertainty reduction is determined by the values of the inputs and assumptions made in the illustration applications presented here, the following conclusions may be broadly relevant to chemical toxicity testing.

1. For a fixed testing time, a greater reduction in uncertainty yields a greater EVDSI. Similarly, for a fixed uncertainty reduction, a reduced testing time results in a greater EVDSI.
2. Because the TRDM cannot take regulatory action based solely on the available prior information in the scenarios considered here, the EVDSI value is always nonnegative, and the CoD never exceeds the EVISI. (If the additional toxicity information does not reduce the uncertainty in the prior information, then the EVDSI would be zero regardless of the time it requires to collect such information.)
3. Because of the trade-offs between timeliness and uncertainty reduction, different combinations of these two factors can produce similar EVDSI values. Under Scenario 1 for an acute outcome, for example, a test that reduces uncertainty about $\mu_{\text{tox}}$ by 70% and requires 5 years to complete has an EVDSI of $31, 145M, whereas another test which only reduces uncertainty about $\mu_{\text{tox}}$ by 40%, but is available almost immediately, has an EVDSI of $31, 357M (Table S2).

**FIGURE 4:**
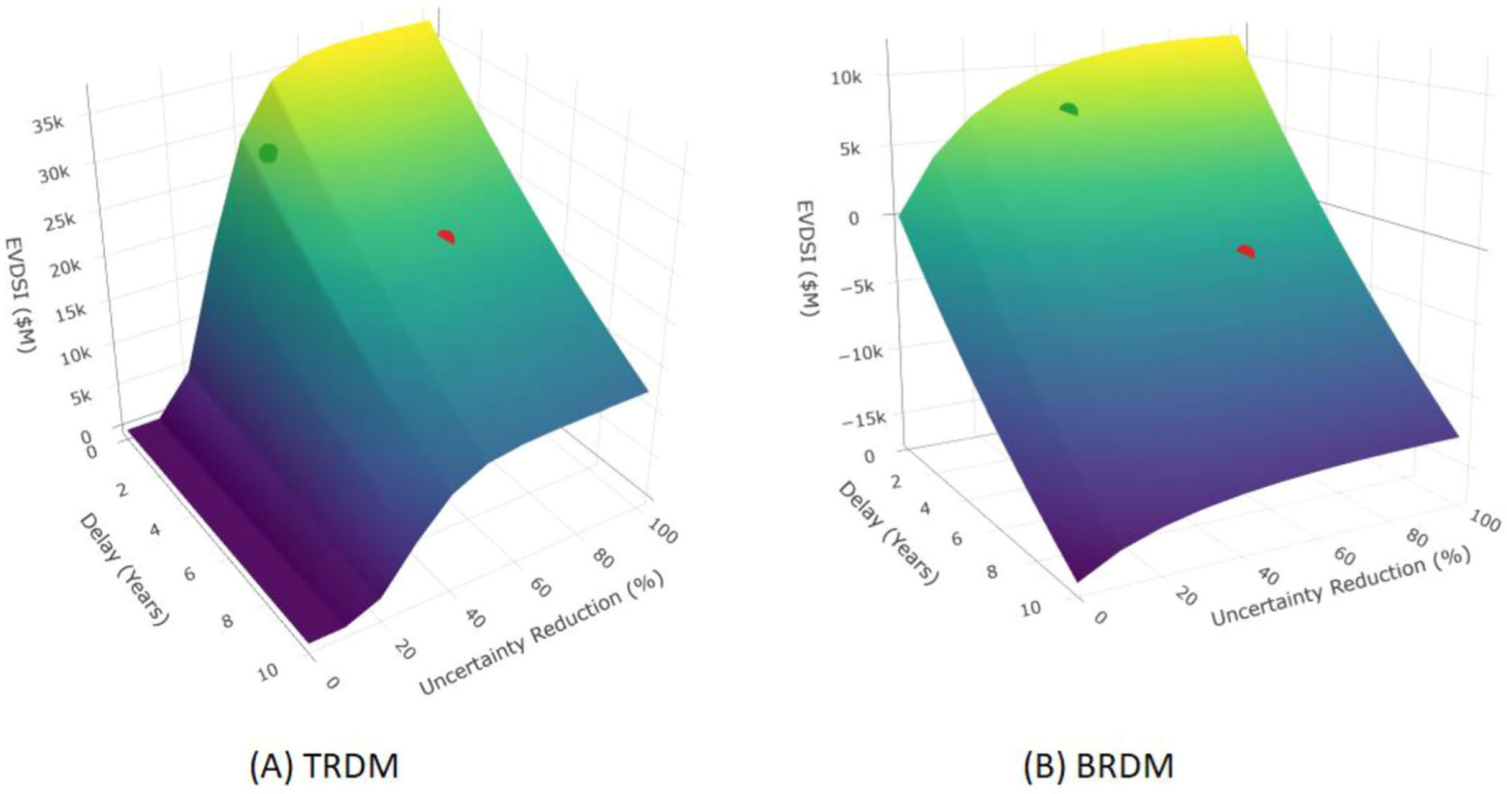
EVDSI for various testing time and uncertainty reduction for a fatal outcome under scenario 1. (A. TRDM; B. BRDM. Green and red spheres represent EVDSI for Tests A and B, respectively.)

Further insights into the trade-offs between timeliness and uncertainty reduction can be seen in the response surfaces for Scenario 1, shown in Figure 4. (Additional response surfaces for TRDM are shown in Figures S1 and S2).

1. Holding the time to collect additional toxicity-testing information constant, the EVDSI increases with the amount of uncertainty reduction. Note, however, that the EVDSI value plateaus with the increase in the reduction in uncertainty.
2. The response surfaces for Scenario 2 (see Figure S1,B) are similar to those for Scenario 1, although the EVDSI values are significantly smaller in Scenario 2 than Scenario 1. In contrast in Scenario 3 and Scenario 1, Scenario 3 does not show the plateauing with increasing uncertainty reduction (Figure. S1–C). This is due to the fact that uncertainty in exposure in Scenario 3 remains unchanged through toxicity testing.

Tables S3 and S4 show the trade-offs between timeliness and uncertainty reduction for the BRDM. As with the TRDM, the BRDM also benefits from increasing EVDSI with increasing uncertainty reduction for a given testing time; for the same level of uncertainty reduction, increased timeliness also leads to increased EVDSI. The BRDM also experiences a balance between timeliness and uncertainty reduction, as was the case for the TRDM. The following unique observation can be made regarding the BRDM.

1. Since the BRDM can make a decision regardless of the amount of uncertainty, the CoD can exceed the EVISI, resulting in a negative EVDSI. In particular, under Scenario 1, even perfect information produces a negative EVDSI (−$2046M) if the information takes 5 years to collect (Table S3). The time required for the CoD to offset the value of additional toxicity information depends on the quality of such information (i.e., the smaller the uncertainty reduction, the more likely it is that the EVDSI would be negative).

### 3.3 | Effect of control cost

To explore the effect of the control cost on VOI for the BRDM (recall that TRDM does not consider control cost), we conducted a series of sensitivity analyses varying the values of these two parameters under the assumptions of Scenario 1 for a fatal outcome. The optimal reduction in exposure depends strongly on the control cost required to reduce the mean population exposure, which was assumed to have a maximum annualized value of ${\text{ACC}}_{\max}=\$2.2\text{B}$ in the illustrative applications discussed above.

Two sensitivity analyses are conducted and summarized in Tables S5 and S6: first we compare the results of VOI analysis under Scenario 1, with the maximum control cost doubled to ${\text{ACC}}_{\max}=\$4.4\text{B}$, or halved to ${\text{ACC}}_{\max}=\$1.1\text{B}$ (Table S5). Since the penalty for overregulation is greatest when ${\text{ACC}}_{\text{max}}$ is largest, the ROI for Test B displays a greater relative improvement (from −454 to 460), than that of Test A (from 1, 391, 550 to 2, 131, 657). On the other hand, when the maximum control cost is halved, the cost of delay has a more significant effect than the annualized control cost, resulting in a less favorable ROI for Test B (−1, 253, compared to −454 in Scenario 1).

The effect of the steepness parameter on the control cost curve, using h=0 and h=4 instead of the default value of h=2 used in Scenario 1, is also studied. When h=0, the control cost is greater than when h>0, and hence Test B shows a greater improvement in ROI (Table S6). Increasing the steepness parameter h has an effect similar to that of reducing the control cost, with more favorable results for both ENBS and ROI when earlier decisions are possible, increasingly favoring Test A as h is increased. The sensitivity analysis on control cost shows that the ROI for Test A is consistently higher than that of Test B, indicating that Test A is uniformly better than Test B with respect to ROI in all analyses conducted here (Table S6).

### 3.4 | Effect of discount rate

In order to explore the effect of discount rates lower than or higher than the U.S. EPA SAB recommendation of 5%, we conducted a sensitivity analysis by setting the discount rate to 3% and 7%, these being the lower and upper limits suggested by the SAB (U.S. EPA, 2004). When the discount rate is reduced to 3%, the ROI for both Tests A and B increased, as future benefits are valued more highly (Table S7). Conversely, when the discount rate is increased to 7%, the ROI for both tests is reduced. These results indicate that the superior performance of Test A compared to Test B is robust to changes in the discount rate within the range of discount rates discussed by the SAB.

### 3.5 | Effect of TRL

In section 3.1, we assumed the TRDM was focused a TRL to be 10^−7^ for the fatal outcome. In order to explore the effects of changing the TRL, we conducted sensitivity analyses in which the TRL is increased to 10^−6^ or 10^−4^ under Scenarios 1 and 3. This analysis indicated that, while the ROI is reduced for both Tests A and B in Scenario 1, the ROI for Test A remain higher than Test B (Table S8). In Scenario 3, on the other hand, The ROI for Test B is actually higher than the ROI for Test A when the TRL is increased to either 10^−6^ or 10^−4^. This results from inability of Test A to sufficiently reduce uncertainty to conclude that exposure mitigation is required. This finding indicates that there may be circumstances in which more expensive longer-term testing resulting in greater uncertainty reduction is beneficial.

## 4 | DISCUSSION AND CONCLUSIONS

In this paper, we present a framework for using measures of VOI to compare and rank different tests that could be used to characterize the toxicity of chemical substances. In its most general form, the methodology described here allows for consideration of the value of the public health benefits derived from risk mitigation actions that may be taken based on the test results. The VOI framework can help in choosing among different toxicity-testing strategies, and considering the trade-offs between timeliness, cost, and uncertainty reduction.

The framework explicitly considers the cost of delay in obtaining additional information (CoD), quantified by the expected value of delayed sample information (EVDSI). The analysis also considers two types of decisionmakers, a BRDM and a TRDM. Although neither decisionmaker fully represents real-world decision-making practices, both decisionmakers follow reasonable decision-making principles, namely balancing risks and benefits and risk minimization (Krewski et al., 2022). An important distinction between the BRDM and TRDM is that while the BRDM is always able to make a decision corresponding to the ORE that minimizes the TSC, even in the presence of large uncertainty, the TRDM is unable to make a decision whenever the uncertainty distribution is such that $q_{05}$ ≤ TRL ≤ $q_{95}$, and must collect additional data before a decision can be taken. In future work, other decision-making rules could also be contemplated, including variations upon the current BRDM and TRDM decision-making approaches, and even hybrid decision-making styles.

Price et al. (2021) conducted a similar analysis comparing alternative toxicity-testing strategies with respective to cost, duration, and uncertainty reduction using cost-effectiveness analysis (CEA). The CEA approach identifies the toxicity-testing strategies with the smallest cost-effectiveness ratio, defined as the cost of testing relative to the probability of making “correct” decisions. Our framework differs from CEA approach in two main ways. First, the present framework evaluates VOI by comparing prior and posterior uncertainties using multiple metrics including ENBS and ROI. This permits us to determine whether any toxicity testing is worthwhile, in addition to determining which of two alternative toxicity-testing approaches is preferable. Second, our framework involves explicit economic valuation of adverse health effect as well as the cost of regulatory intervention. This permits distinguishing between adverse effects of varying severity on an economic basis and valuing the impact of over- and underregulation.

Two illustrative applications, representing a fatal outcome and an acute outcome, comparing two toxicological tests with different characteristics are presented: Test A provides rapid test results at lower cost, but with less reduction in uncertainty than Test B, which requires more time and resources to complete. Our VOI analyses tended to favor Test A over Test B, because both the BRDM and TRDM were able to make more evidence-based decisions earlier, allowing for earlier realization of population health benefits. Comparing scenarios with and without uncertainty in population exposure showed that the value of toxicity testing is reduced in the presence of appreciable uncertainty in exposure, as toxicity testing provides reduced uncertainty in chemical toxicity and not in chemical exposure. Under the assumptions underlying these illustrative applications, our results suggest that less expensive and more rapid toxicological testing strategies can achieve greater overall societal benefit because of the larger numbers of chemicals that can be tested within a fixed budget. Although these assumptions reflect plausible real-world conditions, additional applications involving broader range of conditions would be useful in establishing the extent to which the present findings may be broadly applicable in practice.

It should also be noted that the illustrative applications are based on simplified assumptions. For example, we assumed that the parameters and the sample information follow lognormal uncertainty distributions, which implies that the uncertainty about risk would be right-skewed, and that the presence of uncertainty may lead to overregulation when compared with decisions with perfect information. Another simplifying assumption is that uncertainty only occurs in the measurements of toxicity and exposure. Other sources of uncertainty, such as uncertainty in model structure and the costs of control, can be accommodated within our framework and included in future applications. The assumption of independence among the parameters also greatly simplifies the calculation of the posterior uncertainty distribution but could be relaxed with additional computational effort. Future work extending the present VOI framework should include examination of other distributional assumptions other than lognormal: additional computational effort will be required if nonconjugate prior and posterior distributions are employed.

We are currently pursuing other applications to further gauge the potential contributions of VOI analyses to guide the selection of toxicity-testing strategies in practice. For example, we assumed that the prior uncertainty of an as yet untested chemical could span between five to seven orders of magnitude, and can be reduced by two or more orders of magnitude, depending on the type of testing undertaken. In future work, assumptions about both prior and posterior uncertainty can be refined by examination of inter-chemical variation in the toxic potency of chemicals based on empirical data such as the *in vivo* datasets reported in Krewski et al. (1993a), Thomas, Wesselkamper et al. (2013), Chiu et al. (2018), Gwinn et al. (2020), Johnson et al. (2020), and Pham et al. (2020); the in vitro datasets used by Wetmore et al. (2015), Sand et al. (2017), and Paul-Friedman et al. (2020), and the in silico predictions of toxicity values given in Wignall et al. (2018). As interindividual variability in response to exposure to toxic substances (Zeise et al., 2013) is often the largest source of uncertainty when conducting probabilistic analyses (Chiu et al., 2018), additional work on the relative importance of prior uncertainty in toxicity and exposure would also be valuable. Although the effect of uncertainty in susceptibility was not investigated in this paper, as traditional toxicity testing does not reduce this uncertainty, exploration of this source of uncertainty may be beneficial.

We are also pursuing extension of the analytical framework for VOI analysis proposed here, including evaluating the VOI of different toxicological testing strategies that might be used in regulatory decision making (Krewski et al., 2020; Fantke et al., 2021). These toxicological testing strategies could include sequential or tiered methods of reducing prior uncertainty (Thomas, Philbert et al., 2013), which could be used to refine VOI analyses of the need for further definitive toxicity testing. Other relevant toxicity scenarios worth examining include incorporating information on quantitative structure-activity relationships (QSAR) and classifying chemicals into Cramer classes based on structural alerts (European Food Safety Authority [EFSA], 2019). VOI analyses with chemical classes, such as dioxins and furans or polycyclic aromatic hydrocarbons, could also be informative.

Additional work could also examine the VOI when the target population is partitioned into subgroups based on their vulnerability (e.g., highly sensitive sub-populations or highly exposed occupational populations). The VOI framework can be extended to accommodate multiple adverse effects of different severity associated with exposure to a single agent, which may occur at different levels of exposure (Slikker et al., 2004; Sand et al., 2018). Although the impacts of multiple adverse health outcomes could simply be added as a first approximation, a more complete analysis would involve consideration of competing risks using life-table analyses (Chiang, 1984).

The present paper focuses on VOI analysis for a single agent. To incorporate multiple agents, the VOI methodology presented here will scale in a relatively straightforward manner. This extension is particularly important in chemical risk assessment, where there remain large numbers of chemical substances that have not yet been adequately tested for toxicity (Judson et al., 2009; Hartung, 2017). In the case of untested chemicals subject to equal prior uncertainty, this could be done crudely by scaling the VOI results for a single chemical in proportion to the number of chemicals tested in a long-term testing program. A more refined scaling approach could also consider variable costs such as economies of scale in testing (Andersen et al., 2019; Thomas et al., 2019).

Although health economists are able to value health detriments using approaches such as willingness-to-pay and contingent valuation, the approach for valuing other outcomes is less clear. For example, what is the value of demonstrating a *lack of toxicity* for a specific agent or agents? The BRDM realizes no improvement in health benefit in such cases as a result of toxicity testing, and the TRDM does not need to take risk mitigation action. To address this issue, economists may choose to assign an “option value” (Hanemann, 1989) to negative toxicity test results to recognize the future benefit of knowing that a specific chemical was shown to be safe, though such valuations are outside the scope of the present paper.

In conclusion, this paper presents an analytic framework for determining the value of toxicity-testing information used to support evidence-based risk decision making. Our comprehensive framework for VOI analysis is specifically designed to explore the trade-offs between cost, timeliness, and uncertainty reduction associated with different toxicity-testing methodologies. The framework addresses a number of questions posed by the NRC (2009, p. 90), which recommended “[t]he adoption of formal VOI methods for highly quantified and well-structured decision-making problems, particularly those with very high stakes, clear decision rules, and the possibility of substantial risks associated with delays in decision-making.” One of the most important findings of the analyses (conducted under hypothetical, but plausible, toxicity testing and exposure scenarios) is that timeliness is a strong determinant of VOI. The earlier availability of the toxicity test data to support earlier interventions, if necessary, will result in higher VOI values because the public health benefits of risk mitigation are realized earlier. In almost all of the scenarios considered, the return on investment of less expensive rapid tests was notably greater than that of more expensive test requiring more time to complete, even though the rapid tests produced a smaller reduction in uncertainty. We are continuing efforts to refine the use of VOI methods for toxicity testing, with the concomitant goal of applying our methods to the evaluation of different toxicity-testing strategies under real-world testing conditions.

## Supplementary Material

Supplement1

## Figures and Tables

**TABLE 1:** Parameters common to the illustrative VOI application scenarios

Parameter	Description	Value	Unit
**Toxicity testing information**			
${\text{CoT}}_{\text{A}}$	Cost for Test A	0.005	$M/Test
${\text{CoT}}_{\text{B}}$	Cost for Test B	5	$M/Test
$t_{\text{A}}$	Time to conduct and analyze Test A	1	Years
$t_{\text{B}}$	Time to conduct and analyze Test B	5	Years
**Population and testing information**			
N	Population size	350	Millions of persons
$t_{\text{text}}$ ^1^	Time to analyze test	(0 to 10)	*Years*
$t_{\text{imp}}$	Time to implement regulation	2	Years
$t_{\text{TH}}$	Time horizon	20	Years
r	Discount rate	5	Percent
**Control cost**			
${\text{CC}}_{\text{OH}}$	Overhead cost of regulation	0	$M/Year
${\text{CC}}_{\text{max}}$	Maximum control cost	2,200	$M/Year
η	Steepness of ${\text{CC}}_{\text{k}}$	2	(unitless)

		Value		
Parameter	Description	Fatal	Acute	Unit
**Valuation for health detriment and risk parameters**				
R ^2^	Median risk of prior uncertainty distribution	10^−8^	10^−6^	(unitless)
TRL	Target risk level	10^−7^	10^−5^	(unitless)
V	Economic health cost	8.8 M	50	$/Incident
$B_{y}$	Annualization factor	1/80	365	(unitless)
$\mu_{\text{tox}}$	${\text{log}}_{10}$(${\text{ED}}_{50}$)	1.699	1.699	${\text{log}}_{10}$(mg/kg-bw/day)
$\sigma_{\text{tox}}$	${\text{log}}_{10}$(GSD) of lognormal distribution of individual variability in susceptibility	0.697	0.697	${\text{log}}_{10}$(mg/kg-bw/day)
$\mu_{\exp}$	${\text{log}}_{10}$(median exposure)	−3.115	−2.378	${\text{log}}_{10}$(mg/kg-bw/day)
$\sigma_{\text{exp}}$	${\text{log}}_{10}$(GSD) of lognormal distribution of individual variability in exposure	0.5	0.5	${\text{log}}_{10}$(mg/kg-bw/day)

1 This parameter is used only in the sensitivity analyses and is not required for the main illustrative applications.

2 $R=R_{\mathrm{LT}}$ (lifetime risk) for fatal outcome, and $R=R_{D}$ (daily risk) for acute outcome.

**TABLE 2:** VOI analysis under target-risk decision making

Metric^1^		Fatal Outcome			Acute Outcome		
		Scenario			Scenario		
		1	2	3	1	2	3
**EV|CI ($M)**		44,784	2,839	44,784	62,673	8,359	62,673
**EVIP(P)I ($M)**		36,819	2387	18,069	50,534	6,911	15,706
**EVISI ($M)**	**A**	31,414	183	22	35,377	208	9
	**B**	36,704	2,257	9,886	49,675	5923	7,587
**CoD ($M)**	**A**	2,559	15	2	2,882	17	1
	**B**	13,594	836	3,662	18,398	2,194	2,810
**EVDSI ($M)**	**A**	28,855	168	20	32,495	191	8
	**B**	23,110	1421	6,225	31,277	3,729	4,777
**ENBS ($M)**	**A**	28,855	168	20	32,495	191	7
	**B**	23,105	1,416	6,220	31,272	3,724	4,772
**ROI**	**A**	5,770,902	33,537	4,038	6,498,990	38,294	1,577
	**B**	4,621	283	1,244	6,254	745	954

1 ORE, EV|CI, and EVIPI calculated based on prior information; remaining metrics calculated for Test A and Test B separately.

**TABLE 3:** Probability of making a decision to regulate or not regulate a chemical in different decision contexts

Information		Fatal Outcome			Acute Outcome		
		Scenario			Scenario		
		1	2	3	1	2	3
**TRDM**							
**PTI** ^1^	P(D=1) ^2^	35.4	30.0	1.7	33.5	26.6	1.3
	P(D=0)	64.6	70.0	13.8	66.5	73.4	16.3
	P(D=−1)	0	0	84.5	0	0	82.4
**A**	P(D=1)	5.7	0.1	<0.1	4.8	0.1	<0.1
	P(D=0)	24.9	9.1	0.5	27.6	11.8	0.7
	P(D=−1)	69.4	90.8	99.5	67.6	88.1	99.3
**B**	P(D=1)	18.8	9.6	0.7	17.5	8.1	0.5
	P(D=0)	45.2	43.6	9.3	49.0	47.3	11.0
	P(D=−1)	36.0	46.8	90.0	33.5	44.6	88.5
**BRDM**							
**PTI**	P(D=1)	21.9	14.1	65.9	35.4	30.0	80.9
	P(D=0)	78.1	85.9	34.1	64.6	70.0	19.1
	P(D=−1)	0	0	0	0	0	0
**A**	P(D=1)	47.5	47.0	98.3	65.2	76.9	99.7
	P(D=0)	52.5	53.0	1.7	34.8	23.1	0.3
	P(D=−1)	0	0	0	0	0	0
**B**	P(D=1)	27.5	18.6	75.9	41.5	38.3	87.7
	P(D=0)	72.5	81.4	24.1	58.5	61.7	12.3
	P(D=−1)	0	0	0	0	0	0

1 PTI = perfect toxicity information.

2 D=1, D=0, and D=−1 represent a decision to regulate, a decision not to regulate, or no decision, respectively.

**TABLE 4:** VOI analysis under benefit-risk decision making

Metric^1^		Fatal Outcome			Acute Outcome		
		Scenario			Scenario		
		1	2	3	1	2	3
**ORE (%)**		73	24	73	81	43	81
**EV|CI ($M)**		19,510	2,379	19,510	26,055	5,669	26,055
**EVIP(P)I ($M)**		11,618	1,528	8,405	14,287	3,194	9,811
**EVISI ($M)**	**A**	9,817	744	3,988	11,657	1,466	4,562
	**B**	11,272	1,384	7,529	13,744	2857	8,721
**CoD ($M)**	**A**	2,859	98	2,384	3,933	339	3,355
	**B**	13,536	683	12,149	18,653	2,054	16,792
**EVDSI ($M)**	**A**	6,958	646	1,604	7,724	1,127	1,207
	**B**	−2,263	701	−4,620	−4,909	802	−8,071
**ENBS ($M)**	**A**	6,958	646	1,604	7,724	1127	1,207
	**B**	−2,268	696	−4,625	−4,914	797	−8,076
**ROI**	**A**	1,391,550	129,140	320,840	1,544,750	225,471	241,402
	**B**	−454	139	−925	−983	159	−1,615

1 ORE, EV|CI, and EVIPI calculated based on prior information; remaining metrics calculated for Tests A and B separately.

## Footnotes

1 Points of departures expressed as either no-observed-adverse-effect-levels (NOAELs), lowest-observed-adverse-effect-levels (LOAELs), or lower confidence limit on benchmark dose (BMDL).

2 VSL given in 2016 USD, based on a base value of $7.4M in 2006 USD (U.S. EPA, 2020).
