# Supplementary material for: A value of information framework for assessing the trade-offs associated with uncertainty, duration, and cost of chemical toxicity testing
Source: Risk Anal. Author manuscript; Available in PMC 2024 Mar 1. (PMC10515440; doi:10.1111/risa.13931)
Supplement: Supplement1 [file NIHMS1894119-supplement-Supplement1.pdf]

Supplementary Material

A Value of Information Framework for Assessing the Trade-offs Associated with Uncertainty, Duration, and Cost of Chemical Toxicity Testing

Shintaro Hagiwara,[1,2] Greg M. Paoli,[1] Paul S. Price,[3] Maureen R. Gwinn,[4] Annette Guiseppi-Elie,[3] Patrick J. Farrell,[2] Bryan J. Hubbell,[4] Daniel Krewski,[1,6] and Russell S. Thomas[3]

[1] Risk Sciences International, Ottawa, Canada

[2] School of Mathematics and Statistics, Carleton University, Ottawa, Canada

[3] Center for Computational Toxicology and Exposure, US Environmental Protection Agency, Research Triangle Park, Durham, NC, USA

[4] Sustainable and Healthy Communities Research Program, US Environmental Protection Agency, Research Triangle Park, Durham, NC, USA

[5] Air, Climate, and Energy Research Program, US Environmental Protection Agency, Research Triangle Park, Durham, NC, USA

[6] McLaughlin Centre for Population Health Risk Assessment, University of Ottawa, Ottawa, Canada

In this paper, we have conducted two illustrative VOI analyses (fatal and acute health outcomes) with three hypothetical, but plausible, scenarios to explore the trade-offs between uncertainty reduction and timeliness of toxicity testing.

In addition to the detailed results for the two illustrative applications, sensitivity analyses examining the relationships between the timeliness of toxicity testing ($0, 1, 2, \cdots, 10$ years) and uncertainty reduction ($0\%, 10\%, \cdots, 100\%$ reduction from prior uncertainty) conveniently expressed in response surfaces are presented in this supplementary material. Figs. S1 and S2 display VOI response surfaces for the TRDM with fatal and acute outcomes, respectively; Tables S1 and S2 show the EVDSI values corresponding to these response surfaces. Similarly, Figs. S3 and S4 represent response surfaces for BRDM under the illustrative applications presented in this paper, with Tables S3 and S4 providing these values in a tabular form.

The results of additional sensitivity analyses exploring the effect of control cost ($ACC_{\max} = \$1.1\mathrm{B}$, $\$2.2\mathrm{B}$, and $\$4.4\mathrm{B}$, and $h = 0$, 2, and 4) are summarized in Tables S5 and S6, respectively. Sensitivity analyses of changing the discount rate r (3%, 5%, and 7%) are presented in Table S7. Finally, the results of sensitivity analyses on the TRL ($10^{-7}, 10^{-6}$, and 10^{-4}) are summarized in Table S8.

Response Surface Analysis

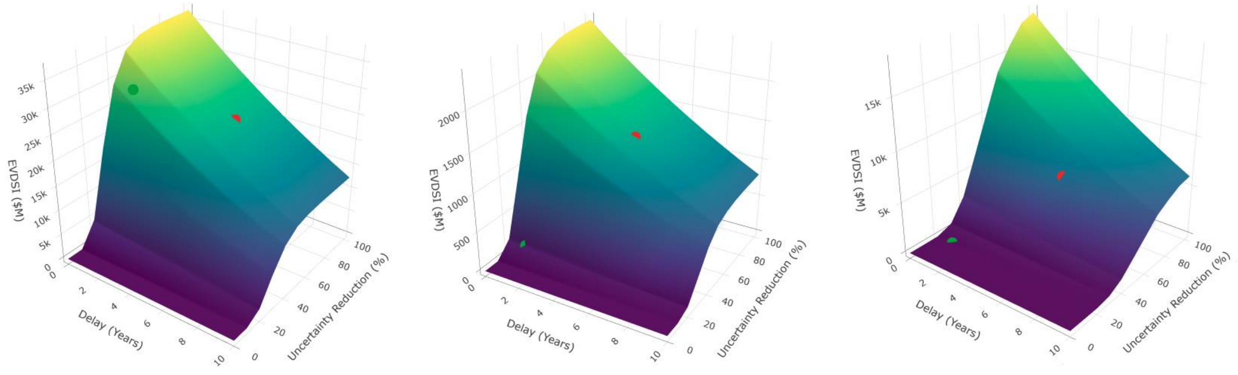

(A) Scenario 1 (B) Scenario 2 (C) Scenario 3

Fig. S1. EVDSI for various testing time and uncertainty reduction for a fatal outcome (TRDM)

Panel (A): Scenario 1, panel (B): Scenario 2, panel (C): Scenario 3. Green and red spheres represent EVDSI for Tests A and B, respectively

Table S1. EVDSI for various testing time and uncertainty reduction for a fatal outcome (TRDM)

Scenario 1		t_{Test} (Years)										
TRDM - Fatal		0	1	2	3	4	5	6	7	8	9	10
Reduction in prior uncertainty about μ_{tox} (%)	100	36,819	33,819	30,963	28,242	25,650	23,182	20,832	18,594	16,462	14,431	12,498
	90	36,814	33,815	30,958	28,238	25,647	23,179	20,829	18,591	16,460	14,429	12,496
	80	36,790	33,793	30,938	28,220	25,630	23,164	20,816	18,579	16,449	14,420	12,488
	70	36,695	33,705	30,858	28,146	25,564	23,104	20,762	18,531	16,406	14,383	12,456
	60	36,215	33,264	30,454	27,778	25,229	22,802	20,490	18,288	16,191	14,194	12,293
	50	34,539	31,725	29,045	26,493	24,062	21,747	19,542	17,442	15,442	13,538	11,724
	40	29,147	26,772	24,510	22,357	20,305	18,352	16,491	14,719	13,031	11,424	9,893
	30	18,069	16,597	15,195	13,860	12,588	11,377	10,224	9,125	8,079	7,082	6,133
	20	4,577	4,204	3,849	3,510	3,188	2,882	2,589	2,311	2,046	1,794	1,553
	10	34	31	28	26	23	21	19	17	15	13	11
	0	0	0	0	0	0	0	0	0	0	0	0

Scenario 2		t_{Test} (Years)										
TRDM - Fatal		0	1	2	3	4	5	6	7	8	9	10
Reduction in prior uncertainty about μ_{tox} (%)	100	2,387	2,193	2,007	1,831	1,663	1,503	1,351	1,205	1,067	936	810
	90	2,383	2,189	2,004	1,828	1,660	1,501	1,349	1,204	1,066	934	809
	80	2,373	2,180	1,995	1,820	1,653	1,494	1,343	1,198	1,061	930	805
	70	2,341	2,151	1,969	1,796	1,631	1,474	1,325	1,182	1,047	918	795
	60	2,257	2,073	1,898	1,731	1,572	1,421	1,277	1,140	1,009	885	766
	50	2,070	1,902	1,741	1,588	1,442	1,303	1,171	1,045	926	811	703
	40	1,605	1,474	1,350	1,231	1,118	1,011	908	811	718	629	545
	30	886	813	745	679	617	558	501	447	396	347	301
	20	183	168	154	140	127	115	103	92	82	72	62
	10	1	1	0	0	0	0	0	0	0	0	0
	0	0	0	0	0	0	0	0	0	0	0	0

Scenario 3		t_{Test} (Years)										
TRDM - Fatal		0	1	2	3	4	5	6	7	8	9	10
Reduction in prior uncertainty about μ_{tox} (%)	100	18,069	16,597	15,195	13,860	12,588	11,377	10,224	9,125	8,079	7,082	6,133
	90	17,461	16,038	14,684	13,393	12,164	10,994	9,879	8,818	7,807	6,844	5,927
	80	15,700	14,421	13,203	12,042	10,937	9,885	8,883	7,928	7,019	6,154	5,329
	70	13,563	12,458	11,405	10,403	9,448	8,539	7,674	6,849	6,064	5,316	4,604
	60	9,886	9,081	8,314	7,583	6,887	6,225	5,594	4,993	4,420	3,875	3,356
	50	6,244	5,736	5,251	4,790	4,350	3,932	3,533	3,153	2,792	2,448	2,120
	40	2,635	2,420	2,216	2,021	1,836	1,659	1,491	1,331	1,178	1,033	894
	30	567	521	477	435	395	357	321	286	254	222	193
	20	22	20	18	17	15	14	12	11	10	9	7
	10	2	2	2	2	1	1	1	1	1	1	1
	0	0	0	0	0	0	0	0	0	0	0	0

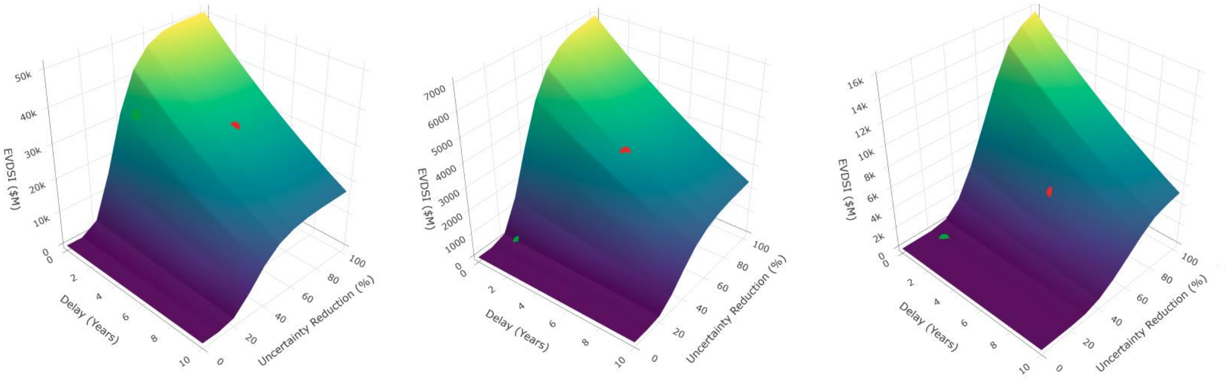

(A) Scenario 1 (B) Scenario 2 (C) Scenario 3

Fig. S2. EVDSI for various testing time and uncertainty reduction for an acute outcome (TRDM)

Panel (A): Scenario 1, panel (B): Scenario 2, panel (C): Scenario 3. Green and red spheres represent EVDSI for Tests A and B, respectively

Table S2. EVDSI for various testing time and uncertainty reduction for an acute outcome (TRDM)

Scenario 1	t_{Test} (Years)										
TRDM - Acute	0	1	2	3	4	5	6	7	8	9	10
Reduction in prior uncertainty about μ_{tox} (%): 100	50,534	46,417	42,496	38,762	35,205	31,818	28,592	25,520	22,594	19,807	17,153
90	50,444	46,334	42,420	38,693	35,142	31,761	28,541	25,474	22,553	19,772	17,123
80	50,248	46,154	42,255	38,542	35,005	31,637	28,430	25,375	22,465	19,695	17,056
70	49,466	45,436	41,598	37,942	34,461	31,145	27,988	24,980	22,116	19,388	16,790
60	47,168	43,325	39,666	36,180	32,860	29,699	26,688	23,820	21,089	18,488	16,011
50	42,083	38,654	35,389	32,279	29,317	26,497	23,810	21,252	18,815	16,495	14,284
40	31,357	28,802	26,369	24,052	21,845	19,743	17,741	15,835	14,019	12,290	10,643
30	15,708	14,428	13,209	12,049	10,943	9,890	8,888	7,933	7,023	6,157	5,332
20	2,990	2,747	2,515	2,294	2,083	1,883	1,692	1,510	1,337	1,172	1,015
10	13	12	11	10	9	8	8	7	6	5	5
0	0	0	0	0	0	0	0	0	0	0	0

Scenario 2	t_{Test} (Years)										
TRDM - Acute	0	1	2	3	4	5	6	7	8	9	10
Reduction in prior uncertainty about μ_{tox} (%): 100	6,911	6,348	5,812	5,301	4,814	4,351	3,910	3,490	3,090	2,709	2,346
90	6,860	6,301	5,769	5,262	4,779	4,319	3,882	3,464	3,067	2,689	2,329
80	6,738	6,189	5,666	5,168	4,694	4,243	3,812	3,403	3,013	2,641	2,287
70	6,464	5,937	5,436	4,958	4,503	4,070	3,657	3,264	2,890	2,534	2,194
60	5,923	5,440	4,981	4,543	4,126	3,729	3,351	2,991	2,648	2,321	2,010
50	4,927	4,525	4,143	3,779	3,432	3,102	2,788	2,488	2,203	1,931	1,672
40	3,312	3,042	2,785	2,540	2,307	2,085	1,874	1,672	1,481	1,298	1,124
30	1,460	1,341	1,228	1,120	1,017	919	826	737	653	572	495
20	208	191	175	160	145	131	118	105	93	82	71
10	0	0	0	0	0	0	0	0	0	0	0
0	0	0	0	0	0	0	0	0	0	0	0

Scenario 3	t_{Test} (Years)										
TRDM - Acute	0	1	2	3	4	5	6	7	8	9	10
Reduction in prior uncertainty about μ_{tox} (%): 100	15,706	14,426	13,207	12,047	10,941	9,889	8,886	7,931	7,022	6,156	5,331
90	15,030	13,806	12,640	11,529	10,471	9,464	8,504	7,590	6,720	5,891	5,102
80	13,822	12,696	11,623	10,602	9,629	8,703	7,820	6,980	6,180	5,417	4,692
70	11,039	10,140	9,283	8,468	7,691	6,951	6,246	5,575	4,936	4,327	3,747
60	7,587	6,969	6,380	5,820	5,286	4,777	4,293	3,832	3,392	2,974	2,575
50	4,237	3,892	3,563	3,250	2,952	2,668	2,397	2,140	1,895	1,661	1,438
40	1,631	1,498	1,371	1,251	1,136	1,027	923	823	729	639	553
30	294	270	247	226	205	185	166	149	132	115	100
20	9	8	7	7	6	5	5	4	4	3	3
10	2	2	1	1	1	1	1	1	1	1	1
0	0	0	0	0	0	0	0	0	0	0	0

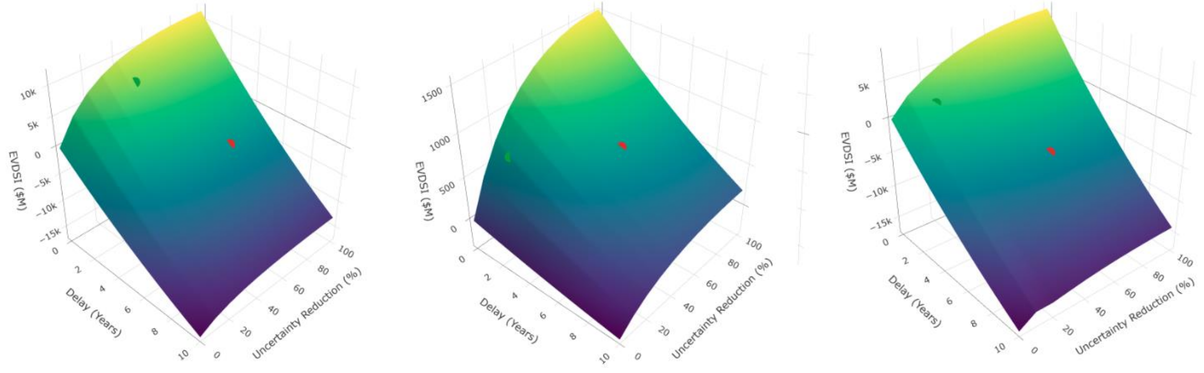

(A) Scenario 1 (B) Scenario 2 (C) Scenario 3

Fig. S3. EVDSI for various testing time and uncertainty reduction for a fatal outcome (BRDM)

Panel (A): Scenario 1, panel (B): Scenario 2, panel (C): Scenario 3. Green and red spheres represent EVDSI for Tests A and B, respectively

Table S3. EVDSI for various testing time and uncertainty reduction for a fatal outcome (BRDM)

Scenario 1		t_{Test} (Years)										
BRDM - Fatal		0	1	2	3	4	5	6	7	8	9	10
Reduction in prior uncertainty about μ_{tox} (%)	100	11,618	8,612	5,750	3,024	427	-2,046	-4,401	-6,644	-8,780	-10,814	-12,752
	90	11,579	8,576	5,717	2,994	400	-2,070	-4,423	-6,663	-8,797	-10,829	-12,765
	80	11,456	8,464	5,614	2,899	314	-2,148	-4,492	-6,725	-8,852	-10,878	-12,807
	70	11,233	8,259	5,426	2,728	159	-2,288	-4,618	-6,838	-8,952	-10,965	-12,882
	60	10,880	7,934	5,129	2,457	-87	-2,511	-4,819	-7,017	-9,110	-11,104	-13,002
	50	10,347	7,445	4,681	2,049	-458	-2,846	-5,120	-7,286	-9,348	-11,312	-13,183
	40	9,560	6,722	4,019	1,445	-1,007	-3,342	-5,565	-7,683	-9,700	-11,621	-13,450
	30	8,403	5,659	3,046	557	-1,813	-4,070	-6,220	-8,267	-10,217	-12,074	-13,843
	20	6,690	4,085	1,605	-757	-3,006	-5,149	-7,189	-9,133	-10,983	-12,746	-14,425
	10	4101	1708	-571	-2742	-4810	-6779	-8654	-10440	-12141	-13760	-15303
	0	0	-2059	-4020	-5888	-7667	-9361	-10974	-12511	-13974	-15368	-16695

Scenario 2		t_{Test} (Years)										
BRDM - Fatal		0	1	2	3	4	5	6	7	8	9	10
Reduction in prior uncertainty about μ_{tox} (%)	100	1,528	1,366	1,212	1,065	925	792	665	544	429	320	215
	90	1,520	1,359	1,205	1,059	920	787	661	540	426	316	213
	80	1,495	1,336	1,184	1,040	902	771	646	528	414	307	204
	70	1,451	1,295	1,147	1,006	871	743	621	505	395	289	189
	60	1,384	1,234	1,091	954	825	701	583	471	365	263	166
	50	1,289	1,146	1,011	881	758	641	530	423	322	226	134
	40	1,158	1,026	900	781	667	559	456	357	264	174	89
	30	981	863	752	645	544	447	355	268	184	105	29
	20	744	646	552	463	379	298	221	148	78	12	-51
	10	428	356	287	221	159	99	43	-11	-63	-112	-158
	0	0	-37	-73	-107	-139	-170	-199	-227	-254	-279	-303

Scenario 3		t_{Test} (Years)										
BRDM - Fatal		0	1	2	3	4	5	6	7	8	9	10
Reduction in prior uncertainty about μ_{tox} (%)	100	8,405	5,661	3,048	559	-1,812	-4,069	-6,219	-8,266	-10,216	-12,074	-13,842
	90	8,354	5,614	3,005	520	-1,847	-4,101	-6,248	-8,292	-10,239	-12,094	-13,860
	80	8,198	5,471	2,873	400	-1,956	-4,199	-6,336	-8,371	-10,309	-12,155	-13,913
	70	7,928	5,223	2,647	193	-2,144	-4,369	-6,489	-8,507	-10,430	-12,261	-14,004
	60	7,529	4,857	2,311	-113	-2,421	-4,620	-6,714	-8,708	-10,608	-12,417	-14,140
	50	6,978	4,351	1,848	-535	-2,805	-4,967	-7,026	-8,986	-10,854	-12,632	-14,326
	40	6,242	3,674	1,229	-1,100	-3,318	-5,430	-7,441	-9,357	-11,182	-12,920	-14,574
	30	5,271	2,784	416	-1,840	-3,988	-6,034	-7,983	-9,839	-11,606	-13,290	-14,893
	20	4,009	1,634	-627	-2,781	-4,832	-6,786	-8,647	-10,419	-12,106	-13,713	-15,244
	10	2444	264	-1811	-3788	-5670	-7463	-9171	-10797	-12346	-13821	-15225
	0	0	-2059	-4020	-5888	-7667	-9361	-10974	-12511	-13974	-15368	-16695

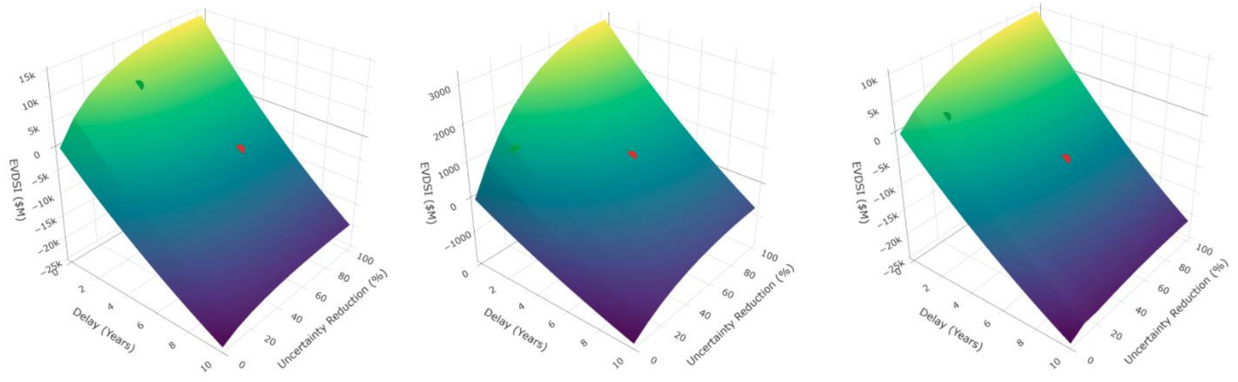

(A) Scenario 1 (B) Scenario 2 (C) Scenario 3

Fig. S4. EVDSI for various testing time and uncertainty reduction for an acute outcome (BRDM)

Panel (A): Scenario 1, panel (B): Scenario 2, panel (C): Scenario 3. Green and red spheres represent EVDSI for Tests A and B, respectively

Table S4. EVDSI for various testing time and uncertainty reduction for an acute outcome (BRDM)

Scenario 1		t_{Test} (Years)										
BRDM - Acute		0	1	2	3	4	5	6	7	8	9	10
Reduction in prior uncertainty about μ_{tox} (%)	100	14,287	10,140	6,190	2,428	-1,155	-4,567	-7,816	-10,911	-13,859	-16,666	-19,339
	90	14,224	10,082	6,137	2,380	-1,198	-4,606	-7,852	-10,943	-13,887	-16,690	-19,360
	80	14,029	9,903	5,973	2,230	-1,334	-4,729	-7,962	-11,041	-13,974	-16,767	-19,427
	70	13,684	9,586	5,683	1,965	-1,575	-4,946	-8,157	-11,216	-14,128	-16,902	-19,544
	60	13,154	9,099	5,237	1,559	-1,944	-5,280	-8,457	-11,483	-14,365	-17,110	-19,724
	50	12,389	8,396	4,594	972	-2,477	-5,762	-8,890	-11,870	-14,707	-17,410	-19,983
	40	11,311	7,406	3,687	146	-3,228	-6,440	-9,500	-12,414	-15,189	-17,832	-20,349
	30	9,809	6,026	2,424	-1,007	-4,275	-7,386	-10,350	-13,173	-15,861	-18,421	-20,859
	20	7,705	4,094	655	-2,621	-5,740	-8,711	-11,540	-14,235	-16,801	-19,246	-21,573
	10	4691	1325	-1880	-4933	-7840	-10609	-13246	-15757	-18149	-20427	-22596
	0	0	-2983	-5825	-8531	-11108	-13562	-15900	-18126	-20246	-22266	-24189

Scenario 2		t_{Test} (Years)										
BRDM - Acute		0	1	2	3	4	5	6	7	8	9	10
Reduction in prior uncertainty about μ_{tox} (%)	100	3,194	2,714	2,258	1,823	1,409	1,014	639	281	-60	-384	-693
	90	3,174	2,696	2,241	1,808	1,395	1,002	628	271	-68	-392	-700
	80	3,115	2,642	2,191	1,762	1,354	965	594	241	-95	-415	-720
	70	3,011	2,547	2,104	1,683	1,282	899	536	189	-141	-456	-755
	60	2,857	2,405	1,974	1,565	1,174	802	448	111	-210	-516	-807
	50	2,642	2,207	1,794	1,400	1,024	667	326	2	-306	-600	-880
	40	2,352	1,941	1,550	1,177	822	484	162	-144	-436	-714	-979
	30	1,968	1,589	1,227	883	555	243	-55	-338	-608	-864	-1,109
	20	1,466	1,127	805	498	205	-73	-339	-591	-832	-1,061	-1,280
	10	816	531	259	-1	-247	-482	-706	-919	-1122	-1316	-1500
	0	0	-219	-428	-627	-816	-996	-1168	-1332	-1487	-1636	-1777

Scenario 3		t_{Test} (Years)										
BRDM - Acute		0	1	2	3	4	5	6	7	8	9	10
Reduction in prior uncertainty about μ_{tox} (%)	100	9,811	6,029	2,426	-1,005	-4,273	-7,385	-10,349	-13,171	-15,860	-18,420	-20,858
	90	9,747	5,969	2,372	-1,055	-4,318	-7,425	-10,385	-13,204	-15,889	-18,445	-20,880
	80	9,550	5,789	2,206	-1,205	-4,455	-7,549	-10,496	-13,303	-15,976	-18,522	-20,947
	70	9,213	5,479	1,923	-1,464	-4,689	-7,761	-10,687	-13,473	-16,127	-18,654	-21,061
	60	8,721	5,028	1,510	-1,841	-5,032	-8,071	-10,965	-13,722	-16,347	-18,847	-21,228
	50	8,052	4,413	946	-2,355	-5,498	-8,492	-11,344	-14,060	-16,646	-19,109	-21,455
	40	7,172	3,604	207	-3,029	-6,111	-9,046	-11,841	-14,503	-17,039	-19,454	-21,753
	30	6,035	2,561	-748	-3,899	-6,900	-9,758	-12,480	-15,072	-17,541	-19,893	-22,132
	20	4,578	1,230	-1,958	-4,994	-7,886	-10,640	-13,263	-15,761	-18,140	-20,406	-22,564
	10	2768	-366	-3350	-6192	-8899	-11477	-13932	-16271	-18498	-20619	-22639
	0	0	-2983	-5825	-8531	-11108	-13562	-15900	-18126	-20246	-22266	-24189

Sensitivity Analysis

Table S2. Sensitivity analysis of changing the maximum control cost for fatal outcome (BRDM – Scenario 1)

Metric[1]		$ACC_{\max} = \$1.1\text{B}$	$ACC_{\max} = \$2.2\text{B}$[2]	$ACC_{\max} = \$4.4\text{B}$
ORE (%)		84	73	61
EV|CI ($M)		15,157	19,510	24,601
EVIP(P)I ($M)		7,708	11,618	16,047
EVISI ($M)	A	6,528	9,817	13,394
	B	7,483	11,272	15,531
CoD ($M)	A	2,946	2,859	2,736
	B	13,744	13,536	13,227
EVDSI ($M)	A	3,582	6,958	10,658
	B	-6,261	-2,263	2,303
ENBS ($M)	A	3,582	6,958	10,658
	B	-6,266	-2,268	2,298
ROI	A	716,455	1,391,550	2,131,657
	B	-1,253	-454	460

[1] ORE, EV|CI, and EVIP(P)I calculated based on prior information; remaining metrics calculated for Test A and Test B separately
[2] $ACC_{\max} = \$2.2B$ is the baseline maximum annual control cost used in Scenario 1 of the main paper.

Table S3. Sensitivity analysis of changing the steepness parameter η on control cost function for fatal outcome (BRDM – Scenario 1)

Metric[1]		$\eta = 0$	$\eta = 2$[2]	$\eta = 4$
ORE (%)		86	73	78
EV|CI ($M)		29,924	19,510	14,276
EVIP(P)I ($M)		21,212	11,618	6,882
EVISI ($M)	A	18,386	9,817	5,771
	B	20,665	11,272	6,670
CoD ($M)	A	2,709	2,859	2,956
	B	13,157	13,536	13,770
EVDSI ($M)	A	15,678	6,958	2,816
	B	7,508	-2,263	-7,099
ENBS ($M)	A	15,677	6,958	2,816
	B	7,503	-2,268	-7,104
ROI	A	3,135,500	1,391,550	563,103
	B	1,501	-454	-1,421

[1] ORE, EV|CI, and EVIP(P)I calculated based on prior information; remaining metrics calculated for Test A and Test B separately
[2] $\eta = 2$ is the baseline steepness parameter for the cost function used in Scenario 1 of the main paper.

Table S4. Sensitivity analysis of changing the discount rate r for the fatal outcome (Scenario 1)

Metric[1]		BRDM			TRDM		
		$r = 3\%$	$r = 5\%^2$	$r = 7\%$	$r = 3\%$	$r = 5\%^2$	$r = 7\%$
ORE (%)		73	73	73	-	-	-
EV|CI ($M)		22,131	19,510	17,453	52,445	44,784	38,795
EVIP(P)I ($M)		13,935	11,618	9,811	44,161	36,819	31,091
EVISI ($M)	A	11,774	9,817	8,290	37,678	31,414	26,527
	B	13,520	11,272	9,519	44,023	36,704	30,994
CoD ($M)	A	2,971	2,859	2,753	2,660	2,559	2,465
	B	14,596	13,536	12,579	14,659	13,594	12,634
EVDSI ($M)	A	8,803	6,958	5,536	35,018	28,855	24,062
	B	-1,076	-2,263	-3,061	29,364	23,110	18,361
ENBS ($M)	A	8,803	6,958	5,536	35,018	28,855	24,062
	B	-1,081	-2,268	-3,066	29,359	23,105	18,356
ROI	A	1,760,634	1,391,550	1,107,292	7,003,660	5,770,902	4,812,462
	B	-216	-454	-613	5,872	4,621	3,671

[1] ORE, EV|CI, and EVIP(P)I calculated based on prior information; remaining metrics calculated for Test A and Test B separately
[2] $r = 5\%$ is the baseline discount rate used in Scenario 1 in Section 3 of the main paper.

Table S5: Sensitivity analysis on TRL for fatal outcome

Metric[1]		Scenario 1	$TRL = 10^{-6}$	$TRL = 10^{-4}$	Scenario 3	$TRL = 10^{-6}$	$TRL = 10^{-4}$
EV|CI ($M)		44,784	44,784	44,784	44,784	44,784	44,784
EVIP(P)I ($M)		36,819	36,797	35,695	18,069	8,983	510
EVISI ($M)	A	31,414	24,585	5,810	22	0	0
	B	36,704	36,194	28,695	9,886	3,637	63
CoD ($M)	A	2,559	2,003	473	2	0	0
	B	13,594	13,405	10,628	3,662	1,347	23
EVDSI ($M)	A	28,855	22,582	5,336	20	0	0
	B	23,110	22,789	18,067	6,225	2,290	39
ENBS ($M)	A	28,855	22,582	5,336	20	0	0
	B	23,105	22,784	18,062	6,220	2,285	34
ROI	A	5,770,902	4,516,382	1,067,245	4,038	73	-13
	B	4,621	4,557	3,612	1,244	457	7

[1] EV|CI, and EVIP(P)I calculated based on prior information; remaining metrics calculated for Test A and Test B separately
